# The Global Burden and Temporal Trends of Psoriasis, 1990–2021: Implications From the COVID‐19 Pandemic

**DOI:** 10.1002/hsr2.73095

**Published:** 2026-08-26

**Authors:** Yunda Guo, Jie Yang, Minglu Xiao, Zhiyan Zhang, Kui Ma, Xiaobing Fu, Siming Yang

**Affiliations:** ^1^ Department of Dermatology The Fourth Medical Center, PLA General Hospital Beijing China; ^2^ The Outpatient Department of 61889 Military Hospital Beijing China; ^3^ Research Center for Tissue Repair and Regeneration Affiliated to the Medical Innovation Research Department, PLA General Hospital and PLA Medical College Beijing China; ^4^ PLA Key Laboratory of Tissue Repair and Regenerative Medicine and Beijing Key Research Laboratory of Skin Injury, Repair, and Regeneration Beijing China; ^5^ Research Unit of Trauma Care, Tissue Repair, and Regeneration Chinese Academy of Medical Sciences Beijing China

**Keywords:** COVID‐19, global burden of disease, joinpoint regression analysis, psoriasis

## Abstract

**Background and Aims:**

Psoriasis can lead to the occurrence of various chronic diseases. However, the detailed reports of psoriasis before and after COVID‐19 are limited. The present study aimed to investigate the global prevalence, incidence, and death and disability adjusted life years (DALYs) of psoriasis with age and sex differences from 1990 to 2021.

**Methods:**

Data were extracted from the Global Burden of Disease (GBD) Study 2021. Our research combined multiple models such as estimated annual percentage change (EAPC), joinpoint regression (JPR), age‐period‐cohort (APC), Bayesian age‐period‐cohort (BAPC) to comprehensively and in‐depth describe the global epidemiological characteristics of psoriasis and its associations with COVID‐19 and respiratory infections.

**Results:**

The ASRs of prevalence, incidence and DALYs were 516, 62, and 44.4 per 100,000 with increased change percentage, 8% (95% UI: 7.7%–8.3%), 8.8% (95% UI: 8.5%–9%) and 8% (95% UI: 7%–9%), respectively. Western Europe, Andean Latin America, and High‐income North America had highest ASRs of prevalence (1155.9, 1018.7, 975.7 per 100,000), incidence (115.3, 105, 103.5 per 100,000), and DALYs (99.8, 88.4, 83.1 per 100,000), while the most changing rate was in East Asia (30.8%, 25.6%, 31%), North Africa and Middle East (27%, 21.4%, 26.5%) and Southeast Asia (20.6%, 16.6%, 20.9%). Moreover, the annual average rate of psoriasis changed significantly before and after the COVID‐19 pandemic with different ages and sex. And the prevalence for psoriasis was negatively correlated with COVID‐19 (*R* = −0.39, *p* < 0.001) and lower respiratory infections (*R* = −0.67, *p* < 0.001). Finally, the trend of psoriasis will keep increasing by 2040.

**Conclusion:**

The prevalence, incidence, and DALYs for psoriasis after COVID‐19 will still increase leading to a severe issue that urgently needs to be solved around world. Our present study integrated with the models and analysis will provide an important theoretical foundation for formulating measures to prevent and treat psoriasis on a global scale in the future.

**Abbreviations**
APC: age‐period‐cohort
ASIR: age‐standardized incidence rates
ASPR: age‐standardized prevalence rates
ASR: age‐standardized rates
BAPC: Bayesian age‐period‐cohort
CI: concentration index
DALY: disability‐adjusted life years
EAPC: estimated annual percentage change
GBD: global burden of diseases
JPR: joinpoint regression
SDI: sociodemographic index
UI: uncertainty interval

## 1 Introduction

Psoriasis serves as a prime example of a prevalent immune‐mediated inflammatory skin condition, affecting 0.09%–11.43% of people worldwide resourced from the World Health Organization, which positions it as one of the most common chronic skin conditions globally [1, 2, 3]. It is not only a frequently encountered chronic skin disorder, typically presenting with distinctive clinical signs including red, scaly patches and persistent itching on the surface of skin [3, 4], but also linked to a diminished quality of life, a shorter life expectancy, and the presence of multiple health issues, such as psoriatic arthritis, cardiovascular diseases and even psychiatric comorbidities, which will lead to large burden among societies and families [5, 6]. Therefore, it is necessary to process a comprehensive epidemiological survey of psoriasis involving all regions and countries globally, as well as various factors.

COVID‐19 is an infection caused by SARS‐CoV‐2 beginning in 2020, which can advance to pneumonia, leading to a more serious or severe manifestation of the disease [7, 8]. As a skin disease caused by immune dysfunction, psoriasis may experience changes in its condition due to systemic infections caused by other diseases. Liu et al. [9] have found that COVID‐19 infection could increase the exacerbation risk in psoriasis lesions and hypertension. Other research also demonstrated that the condition or symptoms of psoriasis have deteriorated after the infection of COVID‐19 [10, 11]. Moreover, some reports indicated that the vaccination of COVID‐19 also caused the exacerbation of psoriasis globally [12, 13]. In contrast, Mendelian randomization studies have shown that there is no definite relationship between the onset of psoriasis and COVID‐19 at the genetic level [14]. In addition, from an epidemiological perspective, the panic about COVID‐19 and the isolation policies may also affect the onset, progression, and subsequent treatment of psoriasis [15].

However, detailed studies and epidemiological characteristics of the associations between psoriasis and COVID‐19 in various regions and countries around the world are still lacking. The Global Burden of Disease (GBD) Study 2021 is the global database of the prevalence, incidence, and death and disability adjusted life years (DALYs) of different diseases for the regions and countries including 5 levels of sociodemographic index (SDI) regions, 21 regions and 204 countries, which can better describe the associations between psoriasis and COVID‐19 geographically [16, 17]. As a result, this study aims to analyze the global burden of psoriasis from 1990 to 2021 using the GBD 2021 data, with a specific focus on trends before and during the COVID‐19 pandemic. We employed estimated annual percentage change (EAPC), joinpoint regression (JPR), age‐period‐cohort (APC), and Bayesian age‐period‐cohort (BAPC) models to identify long‐term patterns, significant trend changes, and the effects of age, period, and cohort. While previous studies may have used one or two of these methods in isolation, our synergistic approach provides a multi‐faceted and deeper understanding. This analysis offers new insights that go beyond most existing analyses.

## 2 Methods

### 2.1 Data Source

Epidemiological data of the present study including psoriasis, COVID‐19, upper and lower respiratory infections from 1990 to 2021 were obtained from the GBD 2021 database. The GBD 2021 database collected the data from GBD study published annually that perform reliable data from various sources around the world to provide coherent projections of health deterioration and specific diseases, which makes the data more rigorous and persuasive so as to more accurately reflect the problems in various regions around the world and assist in formulating relevant policies [18, 19]. Ethical approval with waiver of informed consent was obtained from the University of Washington Institutional Review Board for use of deidentified GBD data.

### 2.2 Data Handling

The present study was performed prevalence, incidence and DALYs data from 1990 to 2021 through the GBD results tool (http://ghdx.healthdata.org/gbd-results-tool) for the following causes: ‘psoriasis’, ‘COVID‐19’, ‘upper respiratory infection’ and ‘lower respiratory infection’. Moreover, we set ‘Global’, ‘All GBD regions’ and ‘All countries and territories’ as locations, “Rate” and “Number” as metrics, ‘All ages’, ‘Age‐standardized’ and ‘All age groups with an interval of 5 years’ as Age groups and ‘Both’, ‘Male’ and ‘Female’ as sex. The selected data were performed by DisMod MR 2.1 (Disease Model, Bayesian Meta‐regression) to compute age‐standardized prevalence rates (ASPRs), age‐standardized incidence rates (ASIRs) and DALYs [20]. The 95% uncertainty interval (UI) was calculated using the 2.5th and 97.5th percentiles of the distribution of the estimates. For the data of the global population from 1990 to 2021, we set “Pop” as measure and “Global” as location and all other options from GBD 2021 database. For the prediction of global population after 2021, these data were harvested from an additional GBD dataset (https://ghdx.healthdata.org/record/ihme-data/global-population-forecasts-2017-21000) [21].

Within the GBD 2021 dataset, cases of psoriasis were defined and identified based on the International Classification of Diseases, Tenth Revision (ICD‐10) code L40. This code corresponds to the broad category of “psoriasis”. While comprehensive for estimating the overall burden of psoriasis, does not systematically differentiate or provide separate estimates for specific clinical subtypes such as plaque psoriasis, guttate psoriasis, or pustular psoriasis. To harmonize the COVID‐19 data across countries, the GBD study conducts a critical and multi‐step process involving several key stages: First, data are collated from a wide array of sources, including official national and subnational surveillance reports, government websites, and open‐access databases. Second, a rigorous data extraction and cleaning procedure is implemented to standardize variables such as case definitions, reporting dates, and demographic classifications, addressing discrepancies in reporting standards. Third, statistical models and cross‐validation techniques are applied to adjust for variations in testing capacities, reporting delays, and differences in diagnostic criteria between countries, thereby creating a consistent and comparable dataset for analysis. This comprehensive approach allows for a more accurate cross‐national comparison of the COVID‐19 burden. All country‐level epidemiological estimates (for both psoriasis and COVID‐19) were utilized directly as age‐standardized values provided by the GBD study, and no re‐standardization was performed by the authors.

### 2.3 Statistical Analysis

Descriptive statistics were performed to describe the number of prevalence and Incidence per millions, ASPR, ASIR, and DALYs among sex, 5 levels SDI regions, 21 GBD regions and 204 countries and territories with UI. R software version 4.4.1 was used to organize the data with tidyverse package. Pearson correlation analysis was used to analyze psoriasis with COVID‐19, and upper and lower respiratory infections with R software version 4.4.1.

EAPC was performed to compare the average rate from 1990 to 2019 and 2019 to 2021 among 21 GBD regions and 204 countries and territories with UI. EAPC is a straightforward statistical method that can be used to analyze trends in a wide range of data [22]. EAPC was calculated by R software version 4.4.1 and the formula of EAPC is shown as follows:

$$\mathrm{EAPC}=\left({\left(\frac{{\mathrm{Rate}}_{\mathrm{final}}}{{\mathrm{Rate}}_{\mathrm{initial}}}\right)}^{\frac{1}{n}}-1\right)\times 100\%.$$

**Figure 1:**
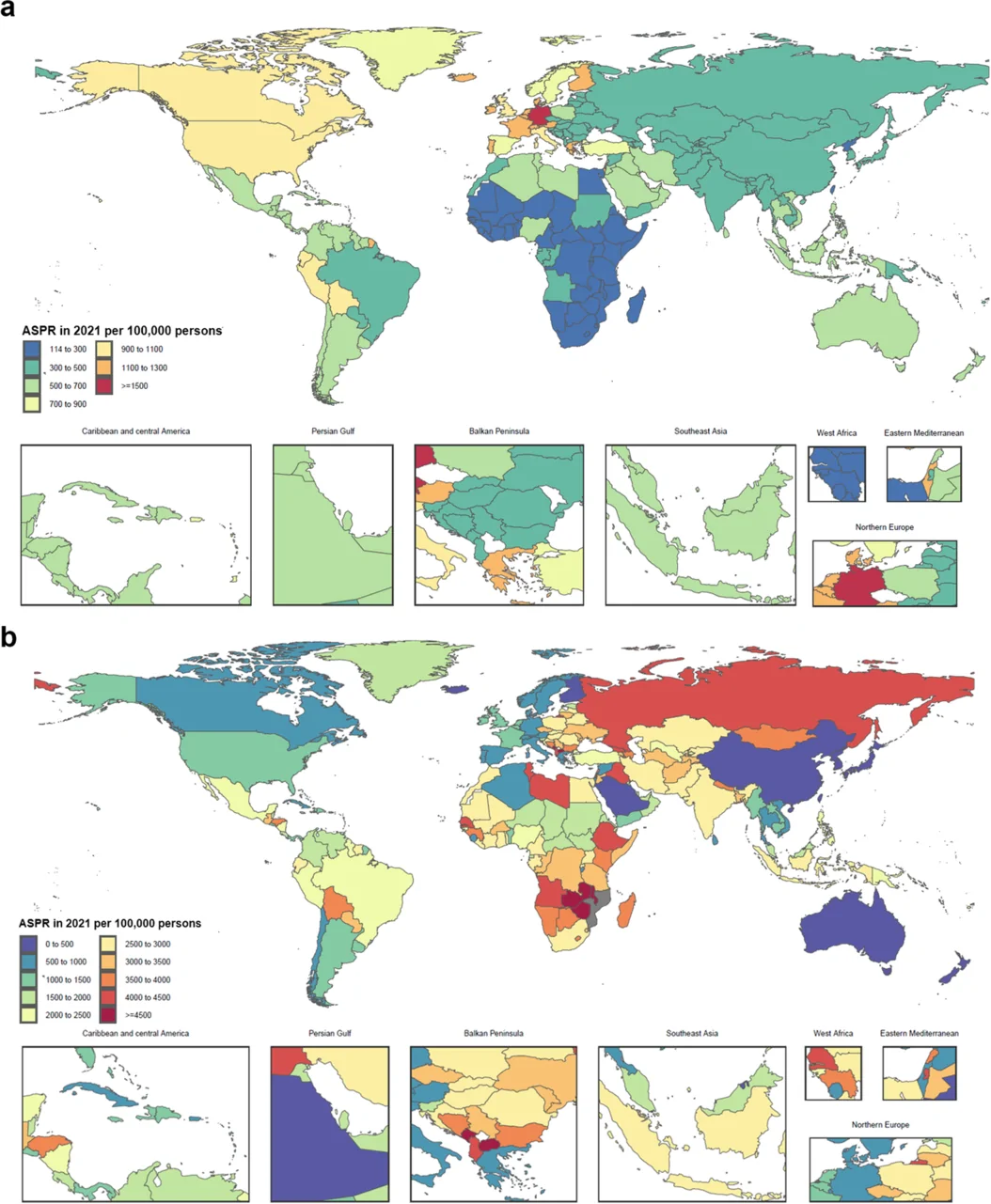
Age‐standardized prevalence of psoriasis and COVID‐19 per 100,000 population by country in 2021. (a) Prevalence ASRs of psoriasis per 100,000 population by country in 2021. (b) Prevalence ASRs of COVID‐19 per 100,000 population by country in 2021.

JPR is applied to identify specific points in time where the trend significantly changes. Unlike EAPC, which assumes a single, consistent trend, JPR detects years where the slope of the trend changes, allowing for a more nuanced analysis of periods with different rates of increase or decrease. JPR model was performed to analyze the significant changes of temporal trends in prevalence, incidence and DALYs from 1990 to 2021 between genders using Joinpoint 5.1.0 software (https://surveillance.cancer.gov/joinpoint). The analysis identified a total of nine joinpoints across the analyzed trends, and their specific temporal positions (years) are clearly indicated in Figure 1.

APC model is employed to disentangle the individual effects of three temporal factors on disease rates: age (the individual's life stage), period (the current calendar year affecting all age groups simultaneously), and cohort (the year of birth, representing generational influences). This is essential for understanding whether observed changes are due to aging, contemporary environmental factors, or the shared experiences of specific birth cohorts. The APC model was performed to estimate the effects of the age, period and cohort with the temporal trends of global prevalence, incidence and DALYs using the APC analysis tool (https://analysistools.cancer.gov/apc/).

At last, BAPC is particularly advantageous for handling data sparsity (e.g., in sub‐group analyses or rare diseases) and producing more stable estimates with credible intervals. The BAPC model was performed to predict the global trend from 2022 to 2040 with R software version 4.4.1 using INLA and BAPC packages. Statistical significance was set at an a priori two‐sided alpha level of 0.05.

## 3 Results

### 3.1 Epidemiological Analysis of Psoriasis From 1990 to 2021

From global level, in 2021, the number of 43 million (95% UI: 41.7–44.3) prevalent cases of psoriasis were reported globally with ASRs 516 (per 100,000), and 5.1 million (95% UI: 4.9–5.3) incidence cases with ASRs 62 (per 100,000). The prevalence and incidence of ASRs change from 1990 to 2021 were 8% (95% UI: 7.7%–8.3%) and 8.8% (95% UI: 8.5%–9%), respectively. Moreover, the DALYs cases were 3689.9 thousand (95% UI: 2684–4917.1) with ASRs 44.4 (per 100,000). The change of DALYs was 8% (95% UI: 7%–9%) (Table 1). Furthermore, the number and ASRs of prevalence (21.7 million, 547.5 per 100,000), incidence (2.6 million, 65.2 per 100,000) and DALYs (1877.4 thousand, 44.4 per 100,000) of male patients in psoriasis was more than that of female patients (21.3 million, 541.9 per 100,000, 2.5million, 64 per 100,000, 1812.5 thousand, 46.1 per 100,000, respectively) (Table 1).

**Table 1:** Prevalent, incidence, and disability adjusted life years (DALYs) for psoriasis in 2021, and percentage change in age standardized rates (ASRs) per 100,000 from 1990 to 2021.

	Prevalence (95% UI)			Incidence (95% UI)			DALYs (95% UI)		
	No., in millions (95% UI)	ASRs per 100,000 (95% UI)	Percentage change in ASRs from 1990 to 2021	No., in millions (95% UI)	ASRs per 100,000 (95% UI)	Percentage change in ASRs from 1990 to 2021	No., in thousands (95% UI)	ASRs per 100,000 (95% UI)	Percentage change in ASRs from 1990 to 2021
Global	43 (41.7, 44.3)	516 (500.2, 531.6)	8 (7.7, 8.3)	5.1 (4.9, 5.3)	62 (60.1, 63.9)	8.8 (8.5, 9)	3689.9 (2684, 4917.1)	44.4 (32.2, 59.2)	8 (7, 9)
Males	21.7 (21, 22.4)	547.5 (530.3, 565.3)	9.6 (9.2, 10)	2.6 (2.5, 2.7)	65.2 (63.2, 67.2)	10.3 (10, 10.7)	1877.4 (1363.7, 2506.6)	47.4 (34.4, 63.3)	9.7 (8.5, 10.9)
Females	21.3 (20.7, 21.9)	541.9 (525.2, 558)	6.6 (6.3, 7)	2.5 (2.4, 2.6)	64 (62.1, 66)	7.3 (7, 7.6)	1812.5 (1320.4, 2412.1)	46.1 (33.6, 61.4)	6.5 (5.3, 7.8)
High SDI	11.5 (11.2, 11.8)	1053.6 (1025.6, 1082.8)	7.5 (6.9, 8.4)	1.1 (1.1, 1.2)	103.7 (100.5, 106.8)	10.2 (9.5, 10.8)	975.4 (714.2, 1297.6)	89.2 (65.3, 118.6)	7.2 (5.6, 8.8)
High‐middle SDI	8.8 (8.5, 9.1)	671.4 (649.3, 694.1)	21.4 (20.9, 21.8)	1 (1, 1)	77.3 (74.8, 79.8)	18.4 (18, 18.9)	750.4 (546.3, 999.4)	57.5 (41.9, 76.6)	21.7 (19.4, 24)
Middle SDI	13.3 (12.8, 13.7)	541.6 (523.3, 559.2)	20.3 (19.9, 20.7)	1.6 (1.6, 1.7)	67 (65, 69.1)	16.9 (16.5, 17.3)	1144.2 (829.6, 1527.3)	46.7 (33.9, 62.4)	20.4 (18.8, 22.1)
Low‐middle SDI	6.9 (6.7, 7.1)	360.4 (348.6, 372)	12 (11.6, 12.5)	0.9 (0.9, 1)	49.3 (47.7, 51)	10.4 (10, 10.9)	599.9 (432.7, 806.8)	31.2 (22.5, 42)	12.4 (9.7, 14.8)
Low SDI	2.5 (2.4, 2.6)	222 (215, 229.3)	8.6 (8, 9.2)	0.4 (0.4, 0.4)	32.6 (31.4, 33.6)	7.9 (7.4, 8.5)	216.7 (157.3, 292.2)	19.4 (14.1, 26.1)	9.2 (5.9, 12.3)
High‐income Asia Pacific	1 (0.9, 1)	395.6 (383.1, 408)	5.3 (4.6, 6.1)	0.7 (−0.1, 1.5)	0.1 (0.1, 0.1)	7.7 (6.9, 8.5)	82.2 (59.7, 109.3)	34.6 (24.9, 46.2)	5.7 (1.8, 9.4)
High‐income North America	4.3 (4.2, 4.4)	975.7 (956.5, 996.6)	4.6 (3.3, 5.9)	0.4 (0.4, 0.4)	103.5 (100.8, 106.1)	7.2 (6, 8.4)	362.2 (265.4, 476.6)	83.1 (60.4, 109.7)	3.4 (1.1, 5.8)
Western Europe	6.4 (6.2, 6.6)	1155.9 (1119.4, 1194.5)	10.9 (10.1, 11.7)	0.6 (0.5, 0.6)	115.3 (111.8, 118.9)	11.2 (10.4, 12)	541.5 (393.9, 723.9)	99.8 (72.2, 133.9)	10.9 (8.6, 13.1)
Australasia	0.2 (0.2, 0.2)	605.3 (583.2, 627.5)	14.9 (13, 16.7)	0 (0, 0)	75.9 (73.2, 78.7)	13.9 (11.9, 16)	18.8 (13.4, 24.9)	52.1 (37.3, 69.6)	15.1 (6.5, 24.1)
Andean Latin America	0.7 (0.6, 0.7)	1018.7 (980.4, 1053.5)	18.7 (17.2, 20.2)	0.1 (0.1, 0.1)	105 (101.1, 109.1)	13.8 (12.2, 15.4)	58.2 (42.2, 77.6)	88.4 (64.1, 117.7)	18.7 (13.4, 24.4)
Tropical Latin America	1 (1, 1)	410.8 (397.9, 424)	−1.1 (−1.7, −0.5)	0.1 (0.1, 0.1)	57.8 (55.9, 59.5)	0.4 (−0.2, 1.1)	85.3 (61.9, 113.2)	35.2 (25.5, 46.8)	−1.1 (−4.4, 2.7)
Central Latin America	1.7 (1.6, 1.7)	644.1 (622.9, 665.3)	10.1 (9.5, 10.6)	0.2 (0.2, 0.2)	78.6 (75.9, 81.2)	8 (7.4, 8.7)	144.9 (104.6, 193.6)	55.6 (40.1, 74.3)	10.1 (7.5, 13)
Southern Latin America	0.5 (0.4, 0.5)	609.5 (587.3, 633.7)	13.8 (12.2, 15.4)	0.1 (0.1, 0.1)	76.9 (73.9, 79.7)	13.5 (11.6, 15.5)	39 (28.4, 52.1)	52.6 (38.3, 70.5)	13.6 (6.6, 20.7)
Caribbean	0.3 (0.3, 0.3)	623.1 (600.8, 646.7)	5.4 (4.4, 6.4)	0 (0, 0)	76.4 (73.5, 79.2)	4.9 (3.8, 5.9)	26.8 (19.2, 35.7)	53.7 (38.4, 71.8)	4.9 (0.8, 9.2)
Central Europe	0.7 (0.7, 0.7)	479.1 (466.2, 491.6)	15.2 (14.1, 16.2)	0.1 (0.1, 0.1)	61.5 (59.8, 63.3)	11.4 (10.5, 12.5)	58.9 (43, 78.1)	41.4 (30, 55.2)	15.5 (12.3, 19.2)
Eastern Europe	1.1 (1.1, 1.2)	442 (427.1, 456.4)	13.8 (13.2, 14.4)	0.1 (0.1, 0.1)	58.6 (56.8, 60.6)	10.9 (10.3, 11.5)	95.2 (69.6, 127.1)	37.9 (27.5, 50.5)	13.7 (10.2, 17.2)
Central Asia	0.4 (0.4, 0.4)	413.7 (398.3, 428.5)	12.1 (11, 13.2)	0.1 (0.1, 0.1)	55.7 (53.6, 57.8)	9.9 (8.7, 11)	34.4 (24.9, 45.8)	35.8 (25.9, 47.8)	12 (6.8, 17.6)
North Africa and Middle East	3.1 (3, 3.2)	508.8 (490.9, 526.4)	27 (26.1, 27.9)	0.4 (0.4, 0.4)	64 (61.8, 66.1)	21.4 (20.6, 22.3)	265.4 (189.4, 355)	43.6 (31.2, 58.3)	26.5 (23.2, 30.2)
South Asia	6.6 (6.4, 6.8)	361 (349.5, 372.4)	8.5 (8.1, 9)	0.9 (0.9, 0.9)	49.4 (47.8, 51)	8.9 (8.5, 9.4)	570.5 (410.8, 765.5)	31 (22.4, 41.7)	9 (6.3, 11.7)
Southeast Asia	3.9 (3.8, 4.1)	545.1 (526.4, 563.2)	20.6 (19.9, 21.2)	0.5 (0.5, 0.5)	67.7 (65.5, 69.8)	16.6 (16, 17.3)	342.9 (249.7, 459.2)	47.2 (34.3, 63.2)	20.9 (17.8, 23.8)
East Asia	8.6 (8.3, 8.9)	466.9 (451.8, 481.5)	30.8 (30.2, 31.5)	1 (1, 1.1)	59.1 (57.2, 60.9)	25.6 (25.1, 26.2)	742.9 (539.1, 990.5)	40.6 (29.4, 54.3)	31 (28.2, 34.4)
Oceania	0 (0, 0)	382.5 (368.7, 396.2)	12.4 (10.9, 14.1)	0 (0, 0)	52.8 (50.6, 54.9)	11.2 (9.4, 13)	3.9 (2.8, 5.2)	32.9 (23.6, 43.6)	12.9 (4.4, 22.6)
Western Sub‐Saharan Africa	1.4 (1.4, 1.5)	366.3 (354.8, 378.5)	15.7 (15.3, 16.1)	0.2 (0.2, 0.2)	42.2 (40.8, 43.5)	12.3 (12, 12.7)	125.8 (91.6, 169.1)	31.6 (22.9, 42.2)	16.3 (13.8, 18.8)
Eastern Sub‐Saharan Africa	0.5 (0.5, 0.5)	150.2 (145.4, 155.1)	3 (2.4, 3.6)	0.1 (0.1, 0.1)	22.9 (22.1, 23.6)	3.2 (2.7, 3.7)	45.3 (33, 60.8)	13 (9.5, 17.3)	3.2 (−1.2, 7.8)
Central Sub‐Saharan Africa	0.3 (0.3, 0.4)	302.9 (292.3, 313.7)	14.6 (12.8, 16.4)	0 (0, 0)	39.3 (37.7, 40.8)	11.9 (10, 13.7)	29.7 (21.6, 40)	26 (18.8, 34.9)	15.7 (5.7, 26.2)
Southern Sub‐Saharan Africa	0.2 (0.2, 0.2)	243.9 (236.2, 252.1)	6.2 (5.5, 6.8)	0 (0, 0)	33.8 (32.6, 34.9)	6.1 (5.3, 6.8)	16.1 (11.6, 21.6)	20.8 (14.9, 27.8)	5.1 (0.4, 10.5)

Abbreviations: ASIR, age‐standardized incidence rates; DALY, disability‐adjusted life years; SDI, sociodemographic index; UI, uncertainty interval.

From GBD regional level, Western Europe, Andean Latin America, and High‐income North America had the highest ASRs of prevalence (1155.9, 1018.7, 975.7 per 100,000), incidence (115.3, 105, 103.5 per 100,000), and DALYs (99.8, 88.4, 83.1 per 100,000) (Table 1). Moreover, the change of prevalence, incidence and DALYs from all regions was increasing from 1990 to 2021 except Tropical Latin America (−1.1%, 0.4%, −1.1%). The regions that have changed the most in the prevalence, incidence and DALYs were concentrated in East Asia (30.8%, 25.6%, 31%), North Africa and Middle East (27%, 21.4%, 26.5%) and Southeast Asia (20.6%, 16.6%, 20.9%) (Table 1).

**Figure 2:**
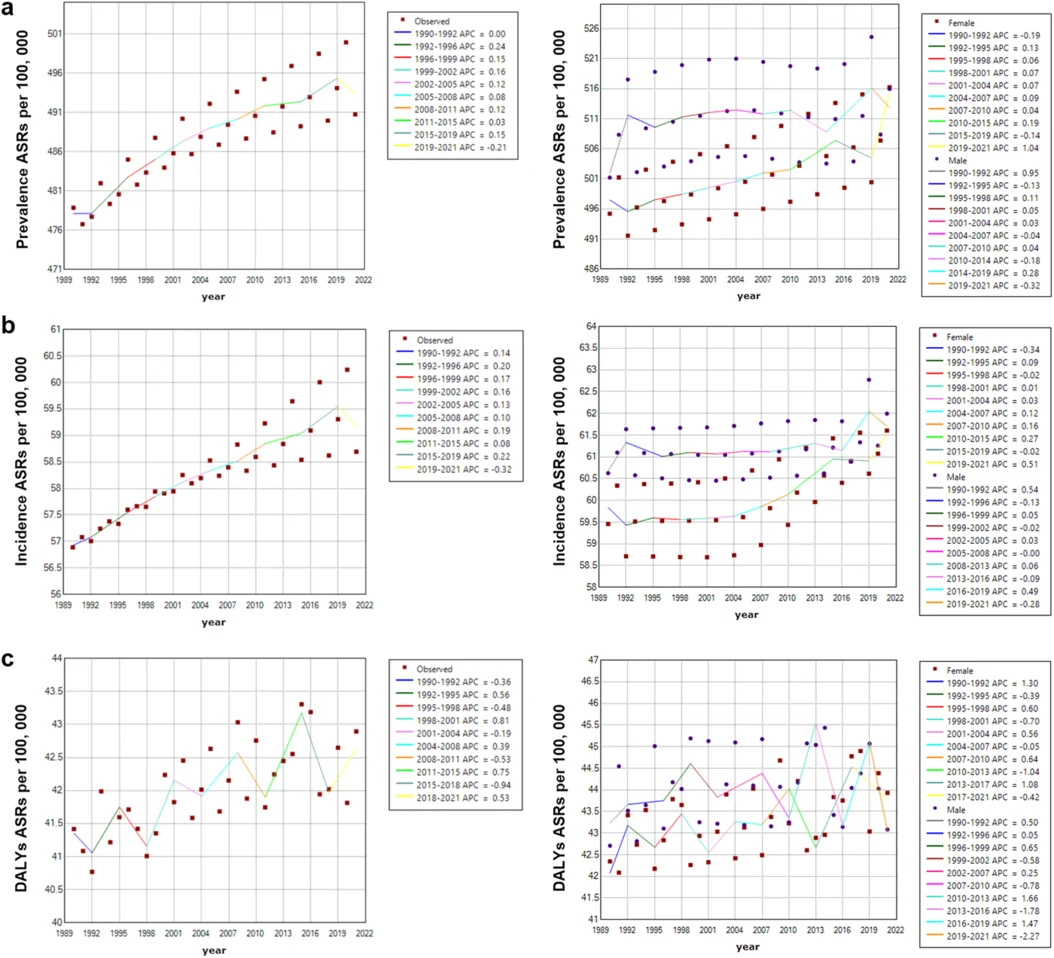
The JPR of prevalence, incidence, and DALYs of psoriasis from 1990 to 2021 in all and between genders. (a) The JPR of prevalence of psoriasis from 1990 to 2021 in all and between genders. (b) The JPR of incidence of psoriasis from 1990 to 2021 in all and between genders. (c) The JPR of DALYs of psoriasis from 1990 to 2021 in all and between genders.

From national level, China (8,453,045), India (5,216,089), and the United States (3,906,305) had the largest prevalence cases of psoriasis in 2021 (Supporting Information S1: Table S1). Moreover, the largest prevalence ASRs were in Germany (1593.7 per 100,000), Switzerland (1299.1 per 100,000) and Monaco (1288.5 per 100,000) (Supporting Information S1: Table S1) (Figure 2a). From 1990 to 2021, the countries with the most significant increase in the percentage ASRs of psoriasis were seen in Singapore (34.5%), Netherlands (32.6%), and Kazakhstan (32.3%) (Supporting Information S1: Table S1).

### 3.2 The Changes in the Average ASRs of Psoriasis Before and After the COVID‐19 Pandemic

From global level, the average rate of prevalence, incidence and DALYs of psoriasis significantly increased from 2019 to 2021 (0.49, 0.64, 0.38) compared with that of psoriasis from 1990 to 2019 (0.23, 0.24, 0.24) measured by EAPC (Table 2). From regional level, the extremely increased change of average rate of prevalence, incidence and DALYs after COVID‐19 pandemic was in South Asia (2.89, 1.91, 2.73), while the extremely decreased change of average rate was in Southern Sub‐Saharan Africa (−0.42, −0.41, −0.63), Western Sub‐Saharan Africa (0.06, −0.2, −0.06) and North Africa and Middle East (0.42, 0.28, 0.27) (Table 2). From national level, the extremely increased change of average rate of prevalence, incidence and DALYs after COVID‐19 pandemic was in India (3.54, 2.34, 3.39), while the extremely decreased change of average rate of prevalence, incidence and DALYs was almost in Mali (−0.99, −0.82, −0.9), Libya (−0.68, −0.36, −0.82), Cabo Verde (−0.66, −0.62, −0.8) (Supporting Information S1: Table S2).

**Table 2:** The average rate of prevalent, incidence, and disability adjusted life years (DALYs) for psoriasis from 1990 to 2019 and from 2019 to 2021 analyzed by EAPC.

	EAPC prevalence (95% UI)		EAPC incidence (95% UI)		EAPC DALYs (95% UI)	
	1990–2019	2019–2021	1990–2019	2019–2021	1990–2019	2019–2021
Global	0.23 (0.22, 0.25)	0.49 (0.06, 0.92)	0.24 (0.22, 0.26)	0.64 (0.14, 1.13)	0.24 (0.22, 0.26)	0.38 (−0.1, 0.85)
High‐income Asia Pacific	0.13 (0.11, 0.15)	0.36 (0.22, 0.5)	0.12 (0.08, 0.17)	1.52 (0.96, 2.08)	0.14 (0.12, 0.16)	0.3 (0.26, 0.35)
High‐income North America	0.15 (0.13, 0.17)	−0.15 (−0.41, 0.11)	0.1 (0.05, 0.15)	1.16 (0.65, 1.68)	0.12 (0.1, 0.14)	−0.33 (−0.52, −0.14)
Western Europe	0.28 (0.24, 0.32)	0.33 (0.29, 0.38)	0.22 (0.16, 0.28)	1.09 (0.38, 1.81)	0.28 (0.24, 0.32)	0.25 (0.13, 0.37)
Australasia	0.61 (0.55, 0.67)	0.31 (0.28, 0.33)	0.43 (0.36, 0.5)	1.34 (0.97, 1.71)	0.61 (0.55, 0.67)	0.24 (0.16, 0.31)
Andean Latin America	0.61 (0.58, 0.63)	0.49 (0.47, 0.51)	0.45 (0.43, 0.47)	0.26 (0.25, 0.27)	0.62 (0.59, 0.64)	0.37 (0.17, 0.57)
Tropical Latin America	−0.06 (−0.08, −0.05)	−0.01 (−0.03, 0.01)	0 (−0.02, 0.02)	−0.06 (−0.13, 0.01)	−0.05 (−0.07, −0.03)	−0.21 (−0.23, −0.18)
Central Latin America	0.31 (0.31, 0.32)	0.23 (0.2, 0.25)	0.25 (0.24, 0.27)	0.09 (0.03, 0.15)	0.32 (0.31, 0.33)	0.09 (0.02, 0.17)
Southern Latin America	0.38 (0.37, 0.39)	0.43 (0.41, 0.46)	0.28 (0.23, 0.33)	1.53 (0.92, 2.14)	0.38 (0.37, 0.39)	0.47 (0.46, 0.48)
Caribbean	0.18 (0.18, 0.19)	0.15 (0.1, 0.2)	0.16 (0.15, 0.17)	0.04 (−0.03, 0.11)	0.18 (0.17, 0.19)	0 (−0.01, 0.01)
Central Europe	0.48 (0.46, 0.5)	0.4 (0.29, 0.52)	0.39 (0.37, 0.42)	0.22 (−0.05, 0.49)	0.5 (0.48, 0.52)	0.29 (0.22, 0.36)
Eastern Europe	0.43 (0.41, 0.46)	0.63 (0.51, 0.74)	0.36 (0.34, 0.39)	0.45 (0.1, 0.81)	0.45 (0.42, 0.48)	0.4 (0.19, 0.61)
Central Asia	0.39 (0.36, 0.42)	0.64 (0.56, 0.71)	0.34 (0.32, 0.37)	0.47 (0.15, 0.79)	0.39 (0.36, 0.42)	0.53 (0.35, 0.71)
North Africa and Middle East	0.84 (0.82, 0.85)	0.42 (0.41, 0.43)	0.68 (0.66, 0.69)	0.28 (0.27, 0.29)	0.83 (0.82, 0.85)	0.27 (0.19, 0.34)
South Asia	0.05 (0, 0.1)	2.89 (−0.06, 5.93)	0.14 (0.12, 0.17)	1.91 (0.22, 3.63)	0.07 (0.03, 0.12)	2.73 (−0.24, 5.8)
Southeast Asia	0.6 (0.59, 0.61)	0.32 (−0.07, 0.71)	0.49 (0.49, 0.5)	0.3 (0.09, 0.52)	0.62 (0.61, 0.63)	0.22 (−0.16, 0.61)
East Asia	0.91 (0.86, 0.96)	0.6 (0.58, 0.63)	0.76 (0.74, 0.79)	0.58 (0.49, 0.68)	0.91 (0.87, 0.96)	0.56 (0.56, 0.57)
Oceania	0.36 (0.34, 0.37)	0.21 (0.06, 0.36)	0.33 (0.32, 0.33)	0.25 (0.11, 0.38)	0.36 (0.35, 0.38)	0.23 (0.11, 0.34)
Western Sub‐Saharan Africa	0.59 (0.54, 0.65)	0.06 (−0.23, 0.35)	0.51 (0.45, 0.56)	−0.2 (−0.37, −0.04)	0.62 (0.57, 0.68)	−0.06 (−0.45, 0.34)
Eastern Sub‐Saharan Africa	0.11 (0.1, 0.13)	−0.06 (−0.4, 0.28)	0.13 (0.11, 0.14)	−0.12 (−0.43, 0.2)	0.14 (0.12, 0.16)	−0.31 (−0.5, −0.13)
Central Sub‐Saharan Africa	0.43 (0.34, 0.52)	0.56 (0.49, 0.63)	0.38 (0.31, 0.45)	0.28 (0.16, 0.4)	0.47 (0.38, 0.57)	0.45 (0.42, 0.48)
Southern Sub‐Saharan Africa	0.28 (0.26, 0.3)	−0.42 (−0.84, −0.01)	0.28 (0.26, 0.3)	−0.41 (−0.66, −0.16)	0.25 (0.23, 0.27)	−0.63 (−0.95, −0.32)

Abbreviations: DALY, disability‐adjusted life years; EAPC, estimated annual percentage change; UI, uncertainty interval.

### 3.3 The JPR of Psoriasis Before and After the COVID‐19 Pandemic

The JPR model showed that the JPR of prevalence and incidence was obviously decreased by −0.21 and −0.32 from 2019 to 2021 (Figure 1a,b), a period including COVID‐19 pandemic, while that of DALYs was extremely increased by 0.53 from 2018 to 2021 (Figure 1c). Furthermore, the JPR of prevalence and incidence in males decreased from 2019 to 2021 (−0.32, −0.28) compared with that of females (1.04, 0.51) (Figure 1a,b). Whilst, the degree of decrease in the JPR of DALYs for males (−2.27) was obviously greater than that of females (−0.28) from 2019 to 2021 (Figure 1c).

### 3.4 The Prediction of Psoriasis From 2022 to 2040

**Figure 3:**
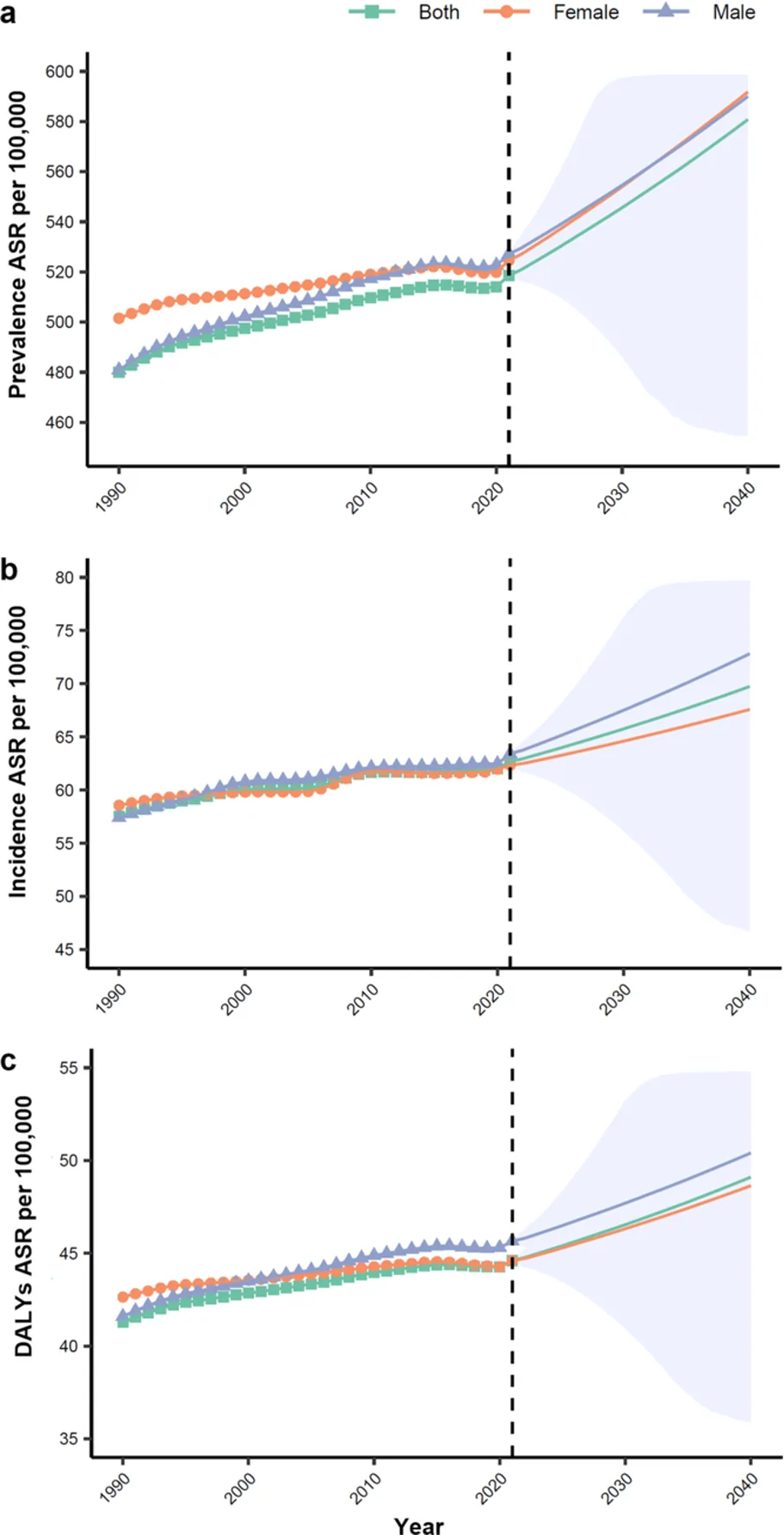
The prediction of prevalence, incidence, and DALYs of psoriasis from 1990 to 2040 in all and between genders measured by BAPC model. (a) The prediction of prevalence of psoriasis from 1990 to 2040 in all and between genders. (b) The prediction of incidence of psoriasis from 1990 to 2040 in all and between genders. (c) The prediction of DALYs of psoriasis from 1990 to 2040 in all and between genders.

The prediction of the trend of psoriasis from BAPC model showed that the global trend of prevalence, incidence and DALYs will continuously increase by 580.7, 69.3, and 49.1 per 100,000 population by 2040 (Figure 3). In addition, the prediction of future trend of incidence (72.8 per 100,000) and DALYs (50.4 per 100,000) in psoriasis of males will still be higher than that of females (67.6, 48.6 per 100,000), while the future trend of prevalence (590 per 100,000) in male showed less than that of female (591.8 per 100,000) by 2040 estimated by BAPC model (Figure 3).

### 3.5 The Distribution of Prevalence of Psoriasis Among Different Age Populations and Sex From 1990 to 2021

**Figure 4:**
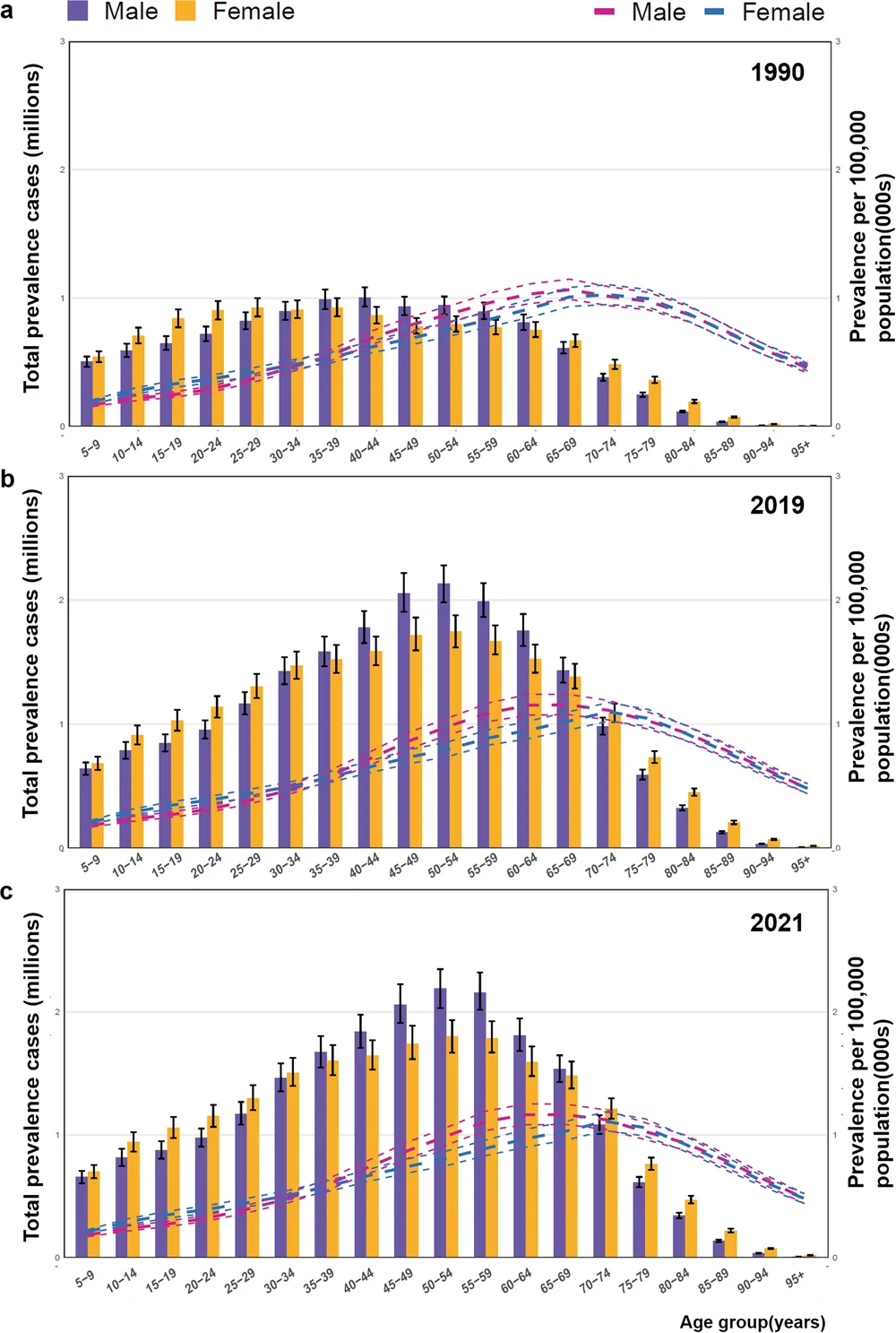
The distribution of prevalence of psoriasis in age groups and sex from 1990 to 2021. (a) The distribution of prevalence of psoriasis in age groups and sex in 1990. (b) The distribution of prevalence of psoriasis in age groups and sex in 2019. (c) The distribution of prevalence of psoriasis in age groups and sex in 2021. Lines indicate prevalent ASRs with 95% uncertainty intervals for men and women. The height of the columns represents the crude prevalence rate. The position of the dashed lines indicates the age‐standardized prevalence rate (ASR) with 95% uncertainty intervals for males and females.

From 1990 to 2021, the prevalence trend of psoriasis had gradually shifted from being mainly concentrated among people aged 35–45 in the early stage to those aged 50–60 (Figure 4). Moreover, compared with the prevalence of psoriasis in 2019, the prevalence of psoriasis among people aged 55–60 increased significantly in 2021 (Figure 4b,c). Furthermore, the number of male patients with psoriasis is higher than that of female patients between the ages of 35 and 70, while the prevalence trend of psoriasis was the opposite among people under 35 years old and those over 70 years old (Figure 4). At last, the mount of prevalence ASRs in males always occurred earlier than that of females (Figure 4).

### 3.6 The Analysis of Prevalence, Incidence, and DALYs for Psoriasis Using APC Model

**Figure 5:**
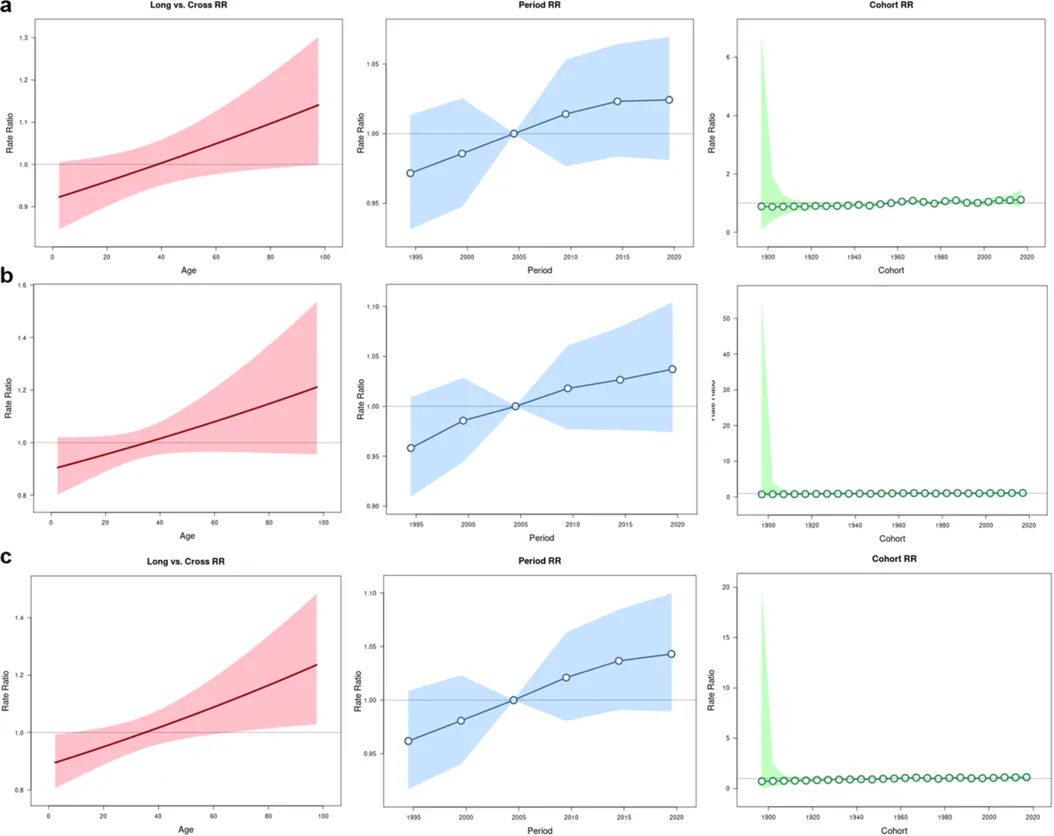
The APC model of prevalence, incidence, and DALYs for psoriasis from 1992 to 2021. (a) The effects of age, period, and cohort for prevalence of psoriasis from 1992 to 2021. (b) The effects of age, period, and cohort for incidence of psoriasis from 1992 to 2021. (c) The effects of age, period, and cohort for DALYs of psoriasis from 1992 to 2021. All rates presented in this figure are crude rates.

To rule out the confounding factors of age and year, and to analyze the impacts of age and year on psoriasis separately, we constructed an APC model. The longitudinal and cross rate ratios of age showed that the prevalence, incidence and DALYs of psoriasis were increasing with age (Figure 5, the first column). Moreover, the period rate ratio showed that the prevalence, incidence and DALYs of psoriasis were increasing with advancing years as well (Figure 5, the second column). At last, the cohort rate ratio showed that with the year of birth to the present, the prevalence, incidence and DALYs of psoriasis were higher.

### 3.7 The Analysis of SDI With Psoriasis Among the 21 Regions and 204 Countries and Territories

**Figure 6:**
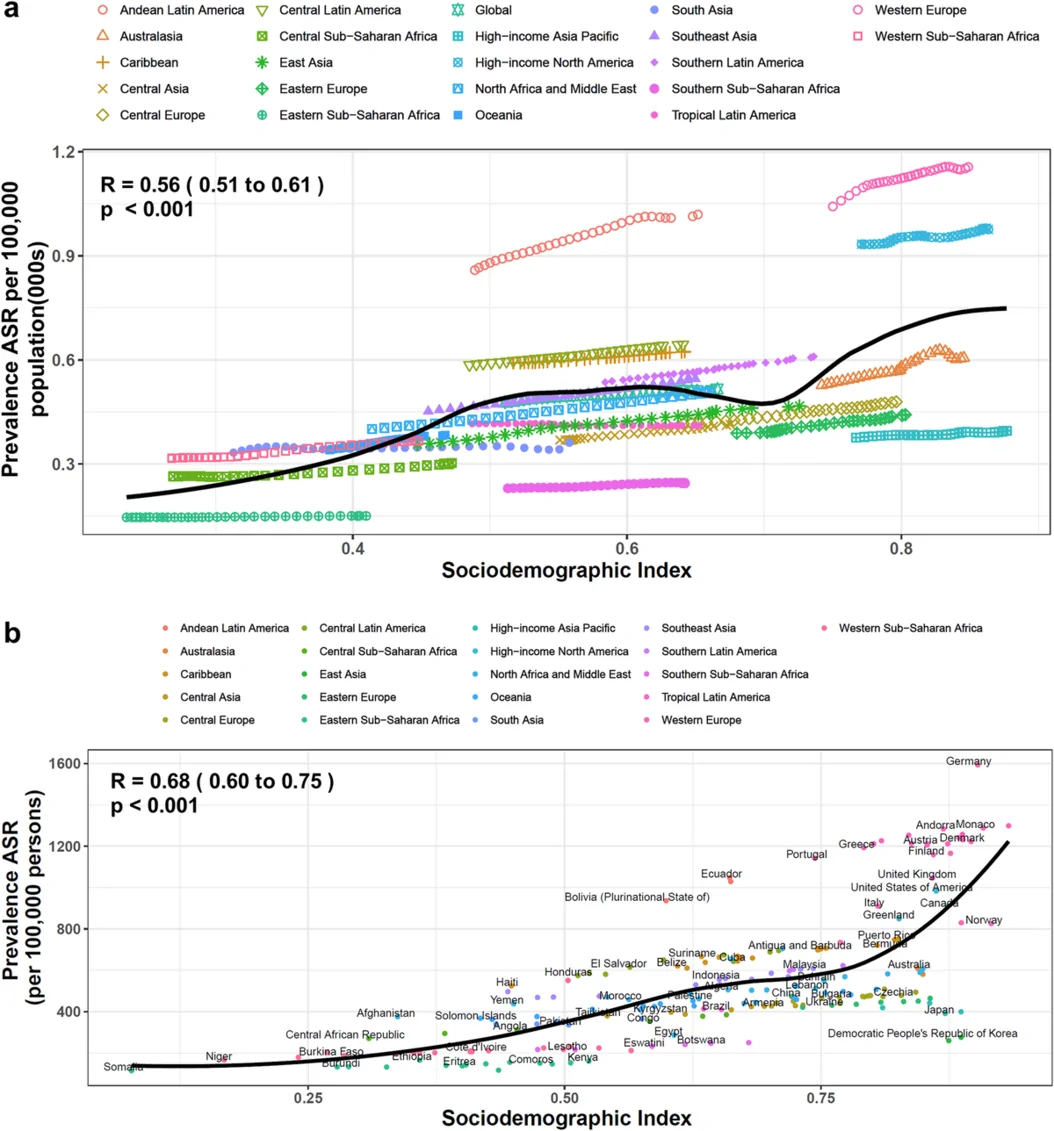
Prevalence ASRs of psoriasis for the 21 GBD regions and 204 countries and territories by SDI. (a) Prevalence ASRs of psoriasis for the 21 GBD regions from 1990 to 2021. Thirty‐two points are plotted for each region and show the observed prevalence ASRs from 1990 to 2021 for that region. (b) Prevalence ASRs of psoriasis for the 204 countries and territories by SDI. Points of different colors represent which region the country was located in.

The highest ASRs of prevalence, incidence and DALYs were in GBD Middle SDI (541.6, 67, 46.7 per 100,000), High‐middle SDI (671.4, 77.3, 54.1 per 100,000) and High SDI (1053.6, 103.7, 89.2 per 100,000) regions (Table 1) in 2021. Whilst, the largest ASRs of change of prevalence, incidence and DALYs were in Middle SDI (21.4, 18.4, 21.7 per 100,000), High‐middle SDI (20.3, 16.9, 20.4 per 100,000) and Low‐middle SDI (12, 10.4, 12.4 per 100,000) regions (Table 1) from 1990 to 2021. Next, we conducted a Pearson correlation analysis on GBD 21 regions and 204 countries with SDI. The SDI of GBD 21 regions was positive correlated with their prevalence ASRs from 1990 to 2021 (*R* = 0.56, *p* < 0.001) (Figure 6a). Moreover, the SDI of 204 countries and territories was also positive with prevalence ASRs (*R* = 0.68, *p* < 0.001), especially the countries of Western Europe.

### 3.8 The Analysis of COVID‐19 and Respiratory Infections With Psoriasis Among 204 Countries and Territories

**Figure 7:**
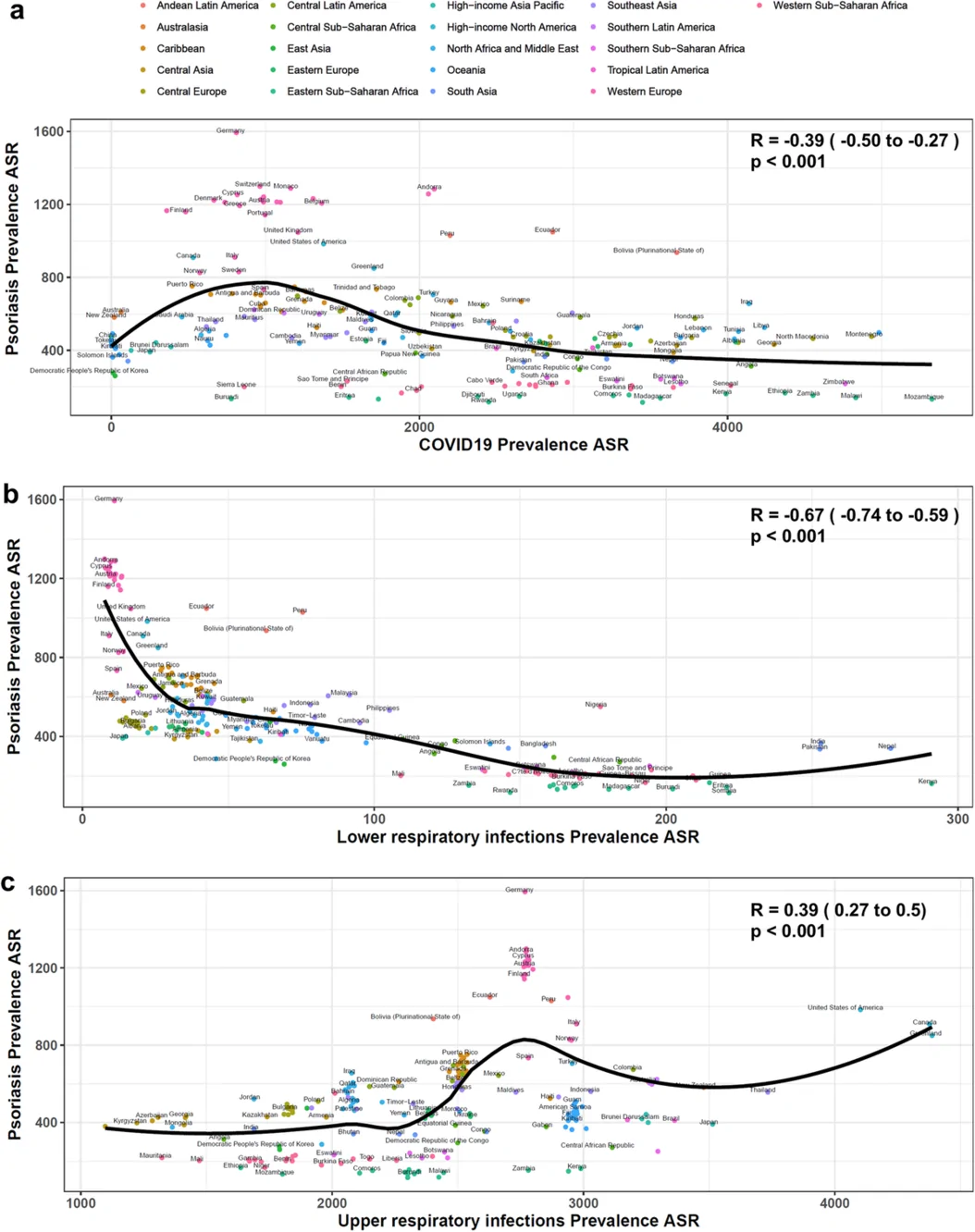
Prevalence ASRs of psoriasis for 204 countries and territories by COVID‐19, lower and upper respiratory infections. (a) Prevalence ASRs of psoriasis for 204 countries and territories by COVID‐19. (b) Prevalence ASRs of psoriasis for 204 countries and territories by COVID‐19 by lower respiratory infections. (c) Prevalence ASRs of psoriasis for 204 countries and territories by COVID‐19 by upper respiratory infections.

From Figure 2, we can identify that prevalence of ASRs was contrast for psoriasis and COVID‐19, especially in some regions of Europe and Africa. To better figure out the relations between psoriasis and COVID‐19, we conducted a Pearson correlation analysis for psoriasis, COVID‐19 and respiratory infections. The analysis showed that the prevalence ASRs of psoriasis were negatively correlated with COVID‐19 (*R* = −0.39, *p* < 0.001) (Figure 7a) and lower respiratory infections (*R* = −0.67, *p* < 0.001) (Figure 7b), while the prevalence ASRs of psoriasis were positive with upper respiratory infections (*R* = 0.39, *p* < 0.001) (Figure 7c).

## 4 Discussion

Psoriasis is a skin disease caused by an immune system disorder and is prevalent worldwide [23, 24]. Although it is a non‐fatal disease, the data collected from GBD showed that the prevalence, incidence and DALYs of psoriasis were becoming more serious all over the world and especially there were obvious changes after COVID‐19 pandemic [15]. These changes may be associated with the COVID‐19 pandemic. Potential explanations for this association could include the potential effects of SARS‐CoV‐2 infection and vaccination on immune function, as well as the psychological and healthcare access disruptions resulting from public health measures such as isolation policies, as suggested by prior research [12, 13, 25]. However, insufficient understanding of epidemiological characteristics may seriously affect clinical treatment and the formulation of relevant policies for psoriasis. As a result, the present study aimed to gain a comprehensive understanding of the global prevalence, incidence and DALYs of psoriasis from 1990 to 2021 and its associations with COVID‐19 and respiratory infections.

In the present study, we utilize the most recent GBD 2021 data and a novel multi‐model analytical framework. This integrated approach provides a more comprehensive, robust, and timely analysis of psoriasis burden trends than previously reported. The GBD 2021 data provide an up‐to‐date foundation, capturing the period through the COVID‐19 pandemic. The multi‐model framework allows us to characterize overall trends, identify significant inflection points, and disentangle the distinct effects of age, period, and cohort, offering deeper insights into the drivers of observed changes. The use of Bayesian APC modeling further enhances the robustness of our estimates. Our data from GBD showed the prevalence, incidence and DALYs of psoriasis has shown an upward trend on a global scale from 1990 to 2021, which was in contrast with the similarly recent study (Its data showed a declined trend worldwide from 1990 to 2019) [4], but consistent with the study(Its data showed an inclined trend worldwide from 1990 to 2017) [26]. The reason for the opposite result may be that there are certain differences between the data collected by GBD 2019 and the GBD 2021 data we applied. The GBD 2019 analysis predates the COVID‐19 pandemic, while our study, utilizing GBD 2021 data, captures trends through 2021. The massive healthcare disruptions, changes in health‐seeking behavior, and potential direct and indirect effects of the pandemic on inflammatory conditions like psoriasis represent a fundamental contextual shift that likely explains a substantial portion of the divergent trends. Moreover, except the updated data we used for analysis of global burden for psoriasis, we also performed EAPC, JPR, APC, BAPC models and Pearson correlation analysis for in‐depth analysis of the impact of age, gender, SDI, and COVID‐19 on psoriasis on GBD 21 regions and 204 countries and territories.

In the present study indicated that regions with high SDI such as Western Europe, Andean Latin America and High‐income North America had the highest prevalence, incidence and DALYs ASRs of psoriasis from 1990 to 2021. From the perspective of ethnic groups, it can be seen that the main ethnic group in these regions is Caucasian, and the genes such as HLA‐Cw6 carried by Caucasian may be more prone to the occurrence of psoriasis [27, 28]. From the perspective of the SDI, compared with other regions, the SDI in these regions is relatively high, so they have more effective diagnostic methods for psoriasis and the public's knowledge about psoriasis, which are relatively overlooked in countries with lower SDI [29]. However, with the development of the era, the SDI in Asia and Africa such as East Asia, North Africa and Middle East and Southeast Asia has been gradually improving, and the largest increase rate of psoriasis already existed in these regions as our results showed, which means Asians may also show characteristics of being prone to psoriasis.

From EAPC model, the average annual growth rate of psoriasis from 2019 to 2021 was significantly higher than that from 1990 to 2019 on global level. This notable acceleration coincides with the COVID‐19 pandemic period. While this temporal association suggests the pandemic may be linked to an exacerbation of psoriasis burden trends, it is crucial to interpret this finding with caution due to the ecological nature of the data, which cannot establish causation. Interestingly, in some GBD regions like South Asia, the burden of psoriasis was obviously aggravated, while in most part of Africa, the burden was significantly reduced. The reason may be influenced by the low medical level in Africa leading to a relatively low diagnosis rate of psoriasis [30, 31]. These remain speculative explanations. A typical example from our study, India of which the change of prevalence, Incidence and DALYs was extremely upward, may reflect that Asians demonstrated a tendency to be prone to psoriasis during COVID‐19 pandemic. Although the global EAPC from 2019 to 2021 was contrast with JPR from our data, the significant time interval was 2019–2021 and the overall upward trend was similar from 1990 to 2021. The overall 3‐year average (EAPC) was pulled positive, likely by a significant increase in burden metrics in 2020 or early 2021. However, the JPR model may have identified a joinpoint followed by a decelerating or declining slopelater in the period (e.g., in 2021). Most epidemiological studies have found that the prevalence of psoriasis is higher in men than in women [32]. Meanwhlie, our results supported that after COVID‐19, the prevalence, incidence and DALYs of psoriasis in male was reduced compared with that of female, of which the age showed a certain degree of delaying trend. Furthermore, the APC model showed that as age, year, and year of birth increase, the global burden of psoriasis will continue to rise, posing significant challenges for the prevention and treatment of psoriasis in the future.

Moreover, the results about the average rate of prevalence, incidence and DALYs of psoriasis before and after the COVID‐19 pandemic indicated that the trend of psoriasis in the world was significantly changed after COVID‐19 pandemic. The results through Pearson correlation analysis showed that the prevalence of psoriasis was positive correlated with SDI and negative with COVID‐19 and lower respiratory infections. Interestingly, while genetic studies have not found a direct link between susceptibility to COVID‐19 and psoriasis [14, 33], our population‐level analysis reveals an ecological association. This discrepancy underscores that the association we observed is unlikely to be driven by shared genetic susceptibility, but rather may stem from broader environmental, behavioral, or healthcare‐system‐related factors concurrent with the pandemic, as discussed above [34]. Additionally, our ecological observation of a negative population‐level association contrasts with some individual‐level clinical reports. While several clinical studies report instances of psoriasis exacerbation or new onset at the individual patient level following infection or vaccination, our ecological analysis at the population level did not detect a corresponding positive association. We clarify that this apparent discrepancy likely stems from the fundamental difference in the level of analysis: clinical studies capture individual biological responses and rare adverse events, whereas ecological studies reflect net population‐level effects that aggregate various competing factors. Ultimately, the results from BAPC model found that the trend of prevalence, incidence and DALYs will still increase for next 20 years, which makes psoriasis a problem that urgently needs to be solved, as psoriasis, an immune‐related skin disease, is usually associated with various primary diseases such as hypertension, diabetes, etc. as well [35].

The data we analyzed in the present research was collected from GBD 2021, which harvested the data from vital registration systems, verbal autopsies, censuses, household surveys, disease‐specific registries, health service contact data, and other sources from all over the world [36]. As a result, the data from GBD 2021 can better reflect the epidemic trends of diseases worldwide. Furthermore, the data integrated with the models and analysis performed by the present research can comprehensively and in‐depth describe the global epidemiological characteristics of psoriasis. Especially, we analyzed the prevalence, incidence and DALYs of psoriasis before and after the COVID‐19 pandemic as well as the association between them, which was not available in other related studies. Beyond its epidemiological insights, our study carries important implications for clinical practice and public health planning. The shifting age and sex patterns identified in our APC analysis, particularly the post‐COVID trends, highlight evolving demographic risk profiles. These findings suggest a need for clinicians, especially in dermatology and primary care, to maintain heightened awareness of psoriasis presentation across a broader age range and among specific birth cohorts. Our identification of regions experiencing the most rapid growth in disease burden can inform more targeted surveillance strategies and resource allocation for diagnostic and therapeutic services in those areas.

This study is subject to several important limitations inherent to its design and data sources. First, our analysis relies on the GBD 2021 dataset, a secondary source with variable data quality and completeness across regions, potentially introducing greater uncertainty in estimates for under‐resourced areas. Second, the ecological nature of our study design is a fundamental consideration. While highly valuable for identifying broad, population‐level trends and generating hypotheses, this approach precludes the establishment of causality or the examination of individual‐level risk factors due to the absence of patient‐level data. Third, our analysis could not stratify findings by psoriatic arthritis, as the current GBD framework does not provide separate estimates for this clinically distinct subtype. Fourth, no statistical correction for multiple comparisons was applied, increasing the risk of Type I error. Finally, the ecological, country‐level associations observed may be confounded by heterogeneous national data on COVID‐19 (e.g., varying testing, reporting, and vaccination rates) and by differences in healthcare access, diagnostic practices, and demographic structures. These factors, alongside potential data inaccuracies in less‐developed regions, limit the mechanistic interpretation of correlations. Our findings thus offer a macro‐level perspective that requires validation through individual‐level clinical and mechanistic studies.

## 5 Conclusion

Our research collected data from GBD 2021 and combined multiple models such as EAPC, JPR, APC, BAPC, along with Pearson correlation analysis to comprehensively and in‐depth describe the global epidemiological characteristics of psoriasis. Moreover, it put forward a hypothetical theory regarding the association with COVID‐19 and respiratory infections, thus providing an important theoretical foundation for formulating measures to prevent and treat psoriasis on a global scale in the future.

## Author Contributions

Yunda Guo, Siming Yang, and Xiaobing Fu contributed to the concept of the manuscript. Yunda Guo, Jie Yang, and Minglu Xiao completed the first draft of the manuscript. Yunda Guo, Jie Yang, Minglu Xiao, Kui Ma, Siming Yang, and Xiaobing Fu contributed to the discussion part of the manuscript. Yunda Guo, Minglu Xiao, and Zhiyan Zhang provided critical feedback on data analysis, results, and discussion. Yunda Guo, Jie Yang, Zhiyan Zhang, and Minglu Xiao managed the estimation process. Yunda Guo, Jie Yang and Siming Yang revised the manuscript critically for important intellectual content. All authors contributed to the framework construction, results interpretation, manuscript revision, and approved the final version of the manuscript. The corresponding authors attest that all listed authors meet authorship criteria and that no others meeting the criteria have been omitted.

## Disclosure

All authors have read and approved the final version of the manuscript. The corresponding author had full access to all the data in this study and takes complete responsibility for the integrity of the data and the accuracy of the data analysis.

## Ethics Statement

The research has been permitted by the institutional review boards of PLA General Hospital. As the present study using historical data, the consent of participants may induce the data selection bias. All privacies of patients were eliminated before data analyses. As a result, the present study was considered lowest risk and the waiver of consent was approved by the institutional review boards of PLA General Hospital.

## Conflicts of Interest

The authors declare no conflicts of interest.

## Transparency Statement

The lead authors, Yunda Guo and Siming Yang, confirm that this manuscript is an honest, accurate, and transparent account of the study being reported; that no important aspects of the study have been omitted; and that any discrepancies from the study as planned (and, if relevant, registered) have been explained.

## Supporting information


Supporting File

## Data Availability

All data generated or analyzed during this study are included in this published article. The data supporting the findings of this study are derived from the Global Burden of Disease Study 2021 (GBD 2021) and are publicly available through the GBD Results Tool (http://ghdx.healthdata.org/gbd-results-tool). The data that support the findings of this study are openly available in the Global Burden of Disease at https://vizhub.healthdata.org/gbd-results/. The authors confirm they had full access to all the data in this study and take complete responsibility for the integrity of the data and the accuracy of the data analysis.
