# Supplementary material for: The Global Burden and Temporal Trends of Psoriasis, 1990–2021: Implications From the COVID‐19 Pandemic
Source: Health Sci Rep. 2026 Aug 26;9(9):e73095. doi: 10.1002/hsr2.73095 (PMC13519089; doi:10.1002/hsr2.73095)
Supplement: Supplementary file 1 [file HSR2-9-e73095-s001.docx]

**Table S1. Prevalence for psoriasis in 1990 and 2021, and percentage change in age standardized rates (ASRs) per 100000 from 1990 to 2021 among 21 regions and 204 countries.**

| **Country** | **1990** | | **2021** | | **Percentage ASRs change per 100000 (1990 to 2021)** |
| --- | --- | --- | --- | --- | --- |
|  | **No (95% UI)** | **ASRs per 100000 (95% UI)** | **No (95% UI)** | **ASRs per 100000 (95% UI)** |  |
| **Global** | 23056630 (22317288,23805091) | 477.7 (462.1,492.7) | 42983446 (41654457,44313231) | 516 (500.2,531.6) | 8 (7.7,8.3) |
| **Andean Latin America** | 277522 (267499,287900) | 858.5 (827.6,889) | 668581 (644024,692168) | 1018.7 (980.4,1053.5) | 18.7 (17.2,20.2) |
| Bolivia (Plurinational State of) | 41232 (39650,42826) | 778.1 (750.5,807.1) | 106476 (102463,110470) | 936.5 (902.6,971.4) | 15 (12.8,17.2) |
| Ecuador | 75928 (73071,78928) | 894.2 (861.2,931.1) | 187642 (180412,194475) | 1048.6 (1008.6,1086.8) | 14.5 (12,16.7) |
| Peru | 160362 (154311,166194) | 864.9 (832.8,895.8) | 374463 (360745,388837) | 1029.5 (992.9,1067.9) | 18.9 (16.7,21.3) |
| **Australasia** | 114112 (110298,118366) | 526.9 (509.3,546.5) | 220556 (212830,228425) | 605.3 (583.2,627.5) | 20.4 (18.1,22.4) |
| Australia | 95560 (92178,99243) | 529.2 (510.7,549.6) | 185587 (178848,192466) | 610.2 (587.8,633) | 17.3 (15,19.6) |
| New Zealand | 18552 (17916,19156) | 515.4 (497.2,531.8) | 34969 (33749,36098) | 581.2 (561.1,602) | 19 (16.9,21.3) |
| **Caribbean** | 191071 (184387,198137) | 591.2 (571.1,614) | 311960 (300625,324248) | 623.1 (600.8,646.7) | 14.9 (13,16.7) |
| Antigua and Barbuda | 360 (347,373) | 632.9 (609.6,656.9) | 705 (679,733) | 703.2 (678.4,729.7) | 15.3 (13.1,17.5) |
| Bahamas | 1583 (1524,1643) | 681.7 (656.5,707.8) | 3042 (2929,3153) | 720.9 (695.3,745.9) | 13.5 (13,13.9) |
| Barbados | 1759 (1692,1824) | 681.9 (655.7,706.8) | 2565 (2463,2659) | 707.6 (679.8,733.3) | 12.8 (11,14.9) |
| Belize | 824 (794,854) | 548.2 (528.1,567.4) | 2538 (2442,2637) | 620.9 (598.4,643.7) | 5.4 (4.4,6.4) |
| Bermuda | 418 (402,434) | 658.8 (634.3,683.1) | 578 (556,600) | 710.8 (684.4,737.5) | 11.1 (9,13.3) |
| Cuba | 67429 (64959,70258) | 619.2 (596.5,644.2) | 88182 (84581,91820) | 644.9 (620.6,668.5) | 12.6 (10.3,15) |
| Dominica | 411 (396,426) | 622.6 (599,645.1) | 522 (502,543) | 698.7 (672,726.8) | 24.4 (21.9,27) |
| Dominican Republic | 32837 (31584,34082) | 542.9 (523.1,563.9) | 67023 (64496,69652) | 611.5 (588.2,635) | 5.7 (3.7,7.9) |
| Grenada | 440 (424,456) | 574.2 (552.6,596.3) | 744 (717,772) | 668.7 (644.4,692.1) | 3.8 (1.8,5.9) |
| Guyana | 3807 (3658,3946) | 583 (561.5,602.5) | 5006 (4815,5211) | 663.4 (638,689.5) | 13.8 (12.2,15.4) |
| Haiti | 25657 (24692,26663) | 485.4 (467.7,503.8) | 60795 (58336,63155) | 524.7 (505.7,544.6) | 13.6 (11.4,15.7) |
| Jamaica | 12703 (12220,13166) | 601.9 (579.1,622.6) | 19614 (18810,20317) | 659.4 (632.4,682.5) | 13.3 (11.2,15.4) |
| Puerto Rico | 24660 (23683,25602) | 678.4 (652.4,704.3) | 30771 (29548,31888) | 752.3 (724.7,778.2) | 7.9 (5.9,10) |
| Saint Kitts and Nevis | 242 (233,251) | 632.1 (608.7,656.4) | 477 (457,497) | 706.9 (678.6,733.5) | 4.1 (2.3,6.1) |
| Saint Lucia | 690 (665,718) | 583.7 (562.4,608) | 1356 (1302,1414) | 661.4 (636.6,687.5) | 12.2 (10.3,14.5) |
| Saint Vincent and the Grenadines | 544 (522,564) | 570.8 (549.9,592.6) | 805 (774,837) | 638.4 (615,663) | 12.6 (10.4,14.9) |
| Suriname | 2153 (2071,2234) | 616.1 (593.1,639.3) | 4101 (3939,4251) | 667.1 (641.5,691.2) | 14 (11.9,16.4) |
| Trinidad and Tobago | 7369 (7088,7656) | 669 (643.7,696.8) | 11758 (11304,12223) | 735.1 (708,762.2) | -1.8 (-3.8,0.3) |
| United States Virgin Islands | 716 (689,742) | 688.9 (664.1,714.9) | 817 (785,850) | 748 (720.1,775.8) | 16.5 (14.2,18.5) |
| **Central Asia** | 223965 (215501,232141) | 369 (355.5,381.5) | 395830 (380915,410263) | 413.7 (398.3,428.5) | 13.8 (11.8,16.3) |
| Armenia | 11891 (11424,12395) | 363.2 (349.1,377.6) | 14941 (14349,15511) | 427.6 (410.9,443.9) | 18.9 (16.5,21.4) |
| Azerbaijan | 25026 (23980,25989) | 378.5 (364,392.8) | 48973 (47055,50914) | 426.3 (410.9,442.1) | 24.2 (21.7,26.8) |
| Georgia | 23893 (22960,24749) | 404.8 (389.1,419.1) | 18394 (17646,19058) | 432 (415,447.5) | 8.1 (5.9,10.3) |
| Kazakhstan | 58022 (55650,60095) | 377 (361.8,390.4) | 83229 (79781,86414) | 429.3 (412.2,445.4) | 32.3 (29.3,34.8) |
| Kyrgyzstan | 14145 (13592,14679) | 368.1 (353.9,382.1) | 25467 (24465,26470) | 397.9 (382.3,413.6) | 24.9 (22.5,27.5) |
| Mongolia | 5701 (5472,5938) | 333.8 (321.4,346.8) | 12281 (11830,12750) | 387.7 (374.4,402.3) | 9.6 (7.5,11.7) |
| Tajikistan | 14616 (14065,15225) | 349.2 (336,362.4) | 34254 (32884,35665) | 380 (365.1,394.3) | 21.8 (21.1,22.6) |
| Turkmenistan | 11220 (10770,11679) | 376.1 (361.8,390.5) | 21380 (20570,22186) | 424.9 (409.1,441.2) | 10.9 (8.7,13.1) |
| Uzbekistan | 59452 (57158,61928) | 351.9 (338.9,365.9) | 136910 (131440,142234) | 409 (392.8,424) | 14.4 (14,14.8) |
| **Central Europe** | 570399 (551116,589745) | 416 (402,430) | 693424 (673933,713912) | 479.1 (466.2,491.6) | 11.8 (9.6,13.8) |
| Albania | 11095 (10666,11489) | 377.1 (363,390.5) | 14315 (13801,14858) | 442.6 (427.6,458.3) | 23.4 (20.9,26.1) |
| Bosnia and Herzegovina | 16918 (16315,17541) | 360.4 (348.2,373.2) | 18933 (18205,19690) | 451 (435.7,466.7) | 13.3 (11,15.6) |
| Bulgaria | 41323 (39833,42848) | 403.9 (389.8,418.5) | 41079 (39536,42739) | 469.7 (453.1,487.5) | 8.5 (8.1,9) |
| Croatia | 23011 (22148,23908) | 412.6 (397.1,427.8) | 25713 (24795,26657) | 478 (461.1,495.8) | 13.8 (11.4,16.1) |
| Czechia | 47779 (46105,49543) | 411.7 (397.3,426.9) | 63896 (61525,66204) | 478.8 (462.1,495.5) | 18.9 (16.4,21.4) |
| Hungary | 48572 (46548,50285) | 404.2 (388,418.4) | 57719 (55383,60003) | 474 (456.6,492.1) | 11.9 (9.8,14.3) |
| Montenegro | 2718 (2622,2818) | 419.6 (405.2,434.4) | 3559 (3431,3692) | 476.2 (459.9,494.1) | 8.3 (6.2,10.3) |
| North Macedonia | 8121 (7820,8405) | 397.2 (382.9,410.7) | 12536 (12071,12990) | 464.6 (448.4,480.6) | 9.9 (7.7,11.9) |
| Poland | 186184 (179873,192446) | 455.8 (440.8,470.5) | 243480 (239071,247325) | 509.1 (500.3,517.1) | 10.3 (8.3,12.5) |
| Romania | 99981 (96133,103911) | 389.3 (374.8,404.9) | 104457 (100751,108522) | 440.1 (424.6,455.8) | 8.6 (6.7,10.5) |
| Serbia | 43488 (41895,45215) | 402.6 (387.9,418.3) | 52586 (50731,54563) | 472.5 (455.6,489.9) | 26.9 (24.2,29.5) |
| Slovakia | 22594 (21814,23391) | 403.5 (389.7,417.7) | 32001 (30815,33264) | 473.4 (456.7,491.6) | 3 (2.4,3.6) |
| Slovenia | 9494 (9148,9858) | 431 (415.4,447.1) | 13055 (12504,13536) | 494.6 (476.1,513.7) | 24.8 (22,27) |
| **Central Latin America** | 807874 (780019,835382) | 585.2 (566,604.5) | 1678611 (1622121,1734179) | 644.1 (622.9,665.3) | 12.1 (11,13.2) |
| Colombia | 170758 (164025,177543) | 606.4 (584.5,629.8) | 352204 (339235,365239) | 674.3 (649.7,699.4) | 3.2 (1.1,5.1) |
| Costa Rica | 16401 (15705,17016) | 624.6 (600.2,647.3) | 35331 (34046,36548) | 688.7 (663.8,712.1) | 6.2 (5.5,6.8) |
| El Salvador | 23925 (23028,24852) | 538 (519.7,556.8) | 39017 (37589,40509) | 615.1 (592.2,638.2) | 3.7 (1.5,6.1) |
| Guatemala | 32440 (31186,33618) | 497 (478.6,516) | 84973 (81720,88100) | 581 (559.4,601.5) | 11.3 (9.2,13.5) |
| Honduras | 18794 (18060,19553) | 507.8 (488.6,527) | 52969 (50877,55009) | 575 (554.2,595.6) | 17.5 (15.3,19.9) |
| Mexico | 417870 (404274,431896) | 583.7 (565,603.1) | 860983 (832430,888522) | 643.3 (622.5,663.3) | 11.4 (9.1,13.9) |
| Nicaragua | 15532 (14885,16180) | 514.8 (495.4,534.7) | 37240 (35799,38665) | 586.4 (564.6,608.6) | 12.6 (10.2,15.1) |
| Panama | 13386 (12886,13913) | 632 (608.7,655) | 30400 (29217,31472) | 695.8 (668.8,720.1) | 4 (1.8,6.3) |
| Venezuela (Bolivarian Republic of) | 98768 (94861,102703) | 617.4 (594.8,640.3) | 185494 (178715,192739) | 650.1 (627.1,674.5) | 4.6 (3.3,5.9) |
| **Central Sub-Saharan Africa** | 114891 (110532,119235) | 264.2 (254.5,273.7) | 340445 (327176,353518) | 302.9 (292.3,313.7) | 17.7 (15.3,20.1) |
| Angola | 20276 (19492,21091) | 249.4 (240.6,258.6) | 80918 (77638,84306) | 313.1 (301.2,324.8) | 19.2 (16.8,21.6) |
| Central African Republic | 5413 (5199,5626) | 246.7 (237.2,256.3) | 12354 (11862,12868) | 270.8 (260.7,282.1) | 12.6 (10.3,15) |
| Congo | 5733 (5504,5948) | 294.9 (283.9,305.3) | 17055 (16346,17724) | 353.4 (339.4,366.8) | 27.2 (26.5,27.9) |
| Democratic Republic of the Congo | 80109 (76837,83182) | 266.4 (256.7,275.8) | 219104 (210292,227526) | 294.7 (283.4,305.2) | 6.7 (4.5,8.7) |
| Equatorial Guinea | 860 (827,895) | 255.2 (245.6,264.8) | 4820 (4609,5032) | 385.5 (371.4,401) | 26.7 (24.2,29.3) |
| Gabon | 2500 (2406,2597) | 301.6 (290.4,313.2) | 6193 (5951,6434) | 378.2 (363.6,392.5) | 11.1 (8.7,13.3) |
| **East Asia** | 4006556 (3871169,4140823) | 356.8 (345.2,368.1) | 8619615 (8324979,8915086) | 466.9 (451.8,481.5) | 13.9 (11.5,16.2) |
| China | 3921863 (3789618,4053307) | 362 (350.3,373.6) | 8453045 (8161389,8743685) | 474 (458.6,488.9) | 28.3 (25.4,31.5) |
| Democratic People's Republic of Korea | 45058 (43445,46781) | 228.8 (220.9,236.9) | 85035 (81743,88016) | 276.2 (266.1,285.6) | 8.1 (5.9,10.5) |
| Taiwan (Province of China) | 39634 (38489,41035) | 202.7 (196.9,209.9) | 81535 (79276,83931) | 260.1 (253.2,267.4) | 16.2 (13.8,18.3) |
| **Eastern Europe** | 973375 (940379,1006453) | 388.5 (375.4,401) | 1126083 (1087577,1166373) | 442 (427.1,456.4) | 8.8 (6.7,11.2) |
| Belarus | 43117 (41425,44836) | 375.1 (360.8,389.5) | 50455 (48379,52343) | 436 (418.4,452.2) | 25.3 (22.8,28) |
| Estonia | 6635 (6369,6908) | 379.4 (365.1,394.3) | 7279 (6986,7567) | 451.3 (434.1,467.9) | 24.4 (21.7,26.8) |
| Latvia | 11560 (11109,12007) | 385.8 (371.4,400) | 10519 (10099,10926) | 448.9 (432.6,465.7) | 13.5 (11.3,15.7) |
| Lithuania | 15417 (14815,16024) | 384 (369,398.8) | 15986 (15334,16608) | 464.8 (446.7,482.1) | 11.2 (9.2,13.3) |
| Republic of Moldova | 16619 (15999,17282) | 364.9 (351,379.2) | 18872 (18037,19600) | 421.3 (403.9,437.7) | 13 (10.5,15.4) |
| Russian Federation | 645876 (624145,667266) | 389.6 (376,401.9) | 788018 (760583,814991) | 445.6 (430.8,459.6) | 16.2 (14,18.8) |
| Ukraine | 234151 (225234,242722) | 390.8 (376.5,404.5) | 234954 (226407,243278) | 431.1 (415.6,446.5) | 15.2 (14.1,16.2) |
| **Eastern Sub-Saharan Africa** | 211936 (204467,219389) | 145.9 (141,150.8) | 512564 (494622,530354) | 150.2 (145.4,155.1) | 17.4 (14.9,19.9) |
| Burundi | 6008 (5794,6231) | 141.1 (136,145.7) | 14023 (13537,14517) | 134.2 (129.5,138.5) | 6.7 (6,7.3) |
| Comoros | 526 (506,546) | 146 (141.1,151) | 1040 (1004,1076) | 151.4 (146.3,156.4) | 14.7 (12.5,16.8) |
| Djibouti | 469 (451,487) | 143.7 (138.4,148.5) | 1675 (1614,1734) | 147 (142,151.8) | -1.2 (-1.9,-0.5) |
| Eritrea | 3556 (3427,3685) | 136.8 (132,141.5) | 7884 (7619,8154) | 142.5 (137.9,147.1) | 23.9 (21.4,26.4) |
| Ethiopia | 60889 (58868,62973) | 158.8 (153.6,163.9) | 144590 (139453,149621) | 165.9 (160.3,171.2) | 18.4 (16.3,20.5) |
| Kenya | 27057 (26164,28014) | 156.6 (151.8,161.6) | 68606 (66368,70928) | 161.5 (156.3,166.5) | 10.2 (8.2,12.4) |
| Madagascar | 13028 (12567,13525) | 140.6 (135.7,145.3) | 32226 (30922,33392) | 138 (133,142.8) | 2.9 (0.9,4.7) |
| Malawi | 10349 (9975,10746) | 138.1 (133.6,143) | 21894 (21058,22715) | 140.5 (135.6,145.5) | 10.9 (8.8,13) |
| Mozambique | 13576 (13098,14094) | 128.4 (124.1,132.7) | 31889 (30781,33073) | 132.5 (128.4,136.9) | 13.7 (11.7,15.6) |
| Rwanda | 6170 (5891,6441) | 112.8 (108.1,117.9) | 13190 (12591,13810) | 117 (111.3,122.1) | 7.3 (5.3,9.4) |
| Somalia | 7402 (7143,7680) | 124.4 (120.6,128.4) | 18561 (17877,19287) | 114.9 (111.1,118.5) | 10 (7.9,12.3) |
| South Sudan | 6445 (6214,6688) | 140 (135.3,144.3) | 10224 (9854,10605) | 132.7 (128.4,137.2) | 7.8 (5.7,9.9) |
| Uganda | 17659 (16986,18305) | 137.7 (133.3,142.3) | 48895 (47230,50711) | 147.8 (142.9,152.6) | 11.2 (9.3,13.7) |
| United Republic of Tanzania | 29914 (28186,31580) | 150.9 (142.6,158.9) | 73731 (69784,77826) | 155.4 (147.4,164.3) | 25.1 (22.6,27.8) |
| Zambia | 8736 (8407,9064) | 146.9 (142.2,152.2) | 23690 (22812,24591) | 152.7 (147.3,157.7) | 12.3 (10,14.4) |
| **High-income Asia Pacific** | 720462 (696722,744388) | 375.6 (363.3,388) | 962513 (931720,993035) | 395.6 (383.1,408) | 16.3 (14.1,18.6) |
| Brunei Darussalam | 834 (803,870) | 387.4 (373.8,402.6) | 1987 (1915,2069) | 419.2 (404.9,435.2) | 15.9 (13.4,18.2) |
| Japan | 557780 (539465,576225) | 382.8 (370.6,394.9) | 660535 (638987,680859) | 391.2 (379,403.4) | 16.3 (14,18.7) |
| Republic of Korea | 149821 (144007,155519) | 350.4 (337.5,363) | 268360 (258317,278480) | 399.6 (385,414) | 17.2 (14.9,19.7) |
| Singapore | 12027 (11545,12491) | 392.4 (378.3,406.4) | 31631 (30398,32813) | 442 (426.6,458.4) | 34.5 (31.9,37.1) |
| **High-income North America** | 2819864 (2735537,2910863) | 933.2 (905.5,961.7) | 4322008 (4229371,4413972) | 975.7 (956.5,996.6) | 13.5 (11.6,15.9) |
| Canada | 239631 (231550,247992) | 810.7 (783,837.6) | 415096 (400829,430451) | 908.8 (877.5,941) | 17 (14.7,19.1) |
| Greenland | 398 (382,414) | 745.2 (718.2,771.8) | 540 (521,560) | 849.6 (821.7,880.5) | 11.7 (9.5,14.4) |
| United States of America | 2579771 (2503078,2662181) | 946.5 (918.7,975.1) | 3906305 (3827071,3984120) | 983.5 (965.1,1003.3) | 13.1 (10.7,15.4) |
| **North Africa and Middle East** | 1078727 (1042464,1114052) | 400.6 (387.1,414.1) | 3076685 (2962329,3188107) | 508.8 (490.9,526.4) | 17.4 (15.3,19.8) |
| Afghanistan | 26140 (25213,27105) | 324.7 (312.8,336.9) | 86155 (82837,89601) | 375.6 (362.5,389.4) | 30.2 (27.4,33) |
| Algeria | 82211 (79042,84994) | 418.5 (402.8,432.6) | 221318 (212325,230300) | 506 (486.6,525.9) | 11.9 (9.9,14.1) |
| Bahrain | 2022 (1938,2103) | 459.2 (441.3,476.3) | 9127 (8715,9529) | 551.1 (529.7,571.6) | 27 (26.1,27.9) |
| Egypt | 106930 (103167,110596) | 234.7 (226.7,242.6) | 274834 (264649,284864) | 286.9 (276.5,297.3) | 15.7 (13,18.4) |
| Iran (Islamic Republic of) | 174295 (168850,179960) | 397.3 (384.6,410.6) | 463717 (447608,479919) | 505.3 (488.9,522.3) | 8.2 (5.9,10.4) |
| Iraq | 68272 (65838,70826) | 503.8 (486,522.7) | 244030 (234192,253650) | 655.7 (629.6,680.8) | 2.2 (1.7,2.8) |
| Jordan | 11943 (11502,12389) | 437.7 (421.8,455) | 60548 (57878,63068) | 521.7 (500.5,542.1) | 14 (11.8,16.2) |
| Kuwait | 7409 (7097,7749) | 494.7 (476.3,513.8) | 30635 (29077,32058) | 589.7 (566.5,611.6) | 9.5 (7.2,11.9) |
| Lebanon | 11119 (10704,11579) | 413.7 (398.2,430.7) | 30176 (29016,31380) | 512.4 (493.7,532) | 14.8 (12.6,17.3) |
| Libya | 13516 (13015,14015) | 420 (404.3,435.4) | 38162 (36532,39806) | 524.8 (505.3,545.5) | 6.2 (5.4,7) |
| Morocco | 79509 (76601,82558) | 380 (365.2,394.3) | 175290 (168441,182548) | 459 (441.5,477.2) | 3.9 (2.6,5.5) |
| Oman | 6527 (6263,6778) | 423 (407,438.8) | 26352 (25162,27590) | 569 (547.4,590.7) | -1.1 (-1.7,-0.5) |
| Palestine | 5382 (5187,5581) | 366.7 (353.5,380.1) | 20252 (19453,21050) | 462.4 (444.5,481.6) | 17.3 (15.1,19.7) |
| Qatar | 1939 (1843,2035) | 476.9 (459.3,494.7) | 19125 (18115,20079) | 592.5 (569.7,616.1) | 1.7 (-0.3,3.7) |
| Saudi Arabia | 53964 (51801,56061) | 440.7 (423.7,457.4) | 231869 (221495,242682) | 583 (561,605.1) | 18 (15.6,20.6) |
| Sudan | 54539 (52489,56446) | 358.8 (345.3,372.2) | 168075 (161916,174218) | 470.7 (453.3,487.8) | 12.8 (12.5,13.2) |
| Syrian Arab Republic | 35357 (34074,36723) | 380.4 (366.4,394.4) | 70002 (67257,72613) | 484.2 (465.7,501.5) | 14.8 (12.2,17.2) |
| Tunisia | 28627 (27597,29702) | 404.8 (390.3,420.4) | 65527 (63049,68234) | 503.6 (484.9,523.1) | 12.1 (10.1,14.1) |
| Turkey | 267838 (257300,277914) | 541.5 (520.8,561.8) | 649200 (623744,674172) | 704.9 (677.8,731.6) | 13.4 (11.3,15.8) |
| United Arab Emirates | 7925 (7563,8306) | 483.9 (467.2,502.4) | 71137 (67098,75082) | 606.6 (583.5,629.9) | 10.1 (9.5,10.6) |
| Yemen | 32673 (31380,33936) | 345.3 (332.6,358.2) | 118284 (113680,122847) | 438.1 (421.8,454.7) | 15.3 (12.8,17.5) |
| **Oceania** | 17187 (16530,17819) | 340.3 (327.2,352.6) | 44638 (42998,46293) | 382.5 (368.7,396.2) | 11.2 (9.2,13.4) |
| American Samoa | 160 (154,166) | 422.8 (406.1,438.8) | 242 (233,251) | 470.3 (452.9,487.9) | 10.3 (8.2,12.3) |
| Cook Islands | 68 (65,70) | 412.2 (396.1,427) | 100 (97,104) | 481.9 (464.9,500.6) | 14.3 (12.2,16.6) |
| Fiji | 2327 (2233,2419) | 377.1 (362.9,392) | 3991 (3843,4140) | 440.6 (424.6,456.5) | 16.3 (14.2,18.5) |
| Guam | 530 (509,550) | 446.2 (429.6,462.6) | 909 (875,943) | 507.8 (488.9,526.2) | 16.9 (14.3,19.2) |
| Kiribati | 211 (204,219) | 362 (349.4,375.9) | 434 (417,450) | 411.2 (396.6,426.5) | 14.1 (11.9,16.6) |
| Marshall Islands | 113 (109,118) | 357.2 (343.8,371.1) | 211 (203,219) | 410.2 (395.1,425.7) | 21.1 (18.7,23.2) |
| Micronesia (Federated States of) | 286 (276,297) | 369.7 (356.6,383.8) | 410 (395,426) | 425.3 (409.5,441.1) | 31.2 (28,33.9) |
| Nauru | 32 (30,33) | 399.1 (384.9,415.1) | 41 (40,43) | 452.5 (435.9,470.7) | 6.7 (4.4,8.8) |
| Niue | 8 (8,9) | 389.2 (374.4,404.1) | 9 (8,9) | 459.2 (441.5,476.3) | 30.2 (27.1,33.1) |
| Northern Mariana Islands | 179 (172,187) | 462.8 (446.2,480.1) | 273 (262,283) | 496.8 (477.7,514.4) | 13.2 (11,15.6) |
| Palau | 58 (56,61) | 431.2 (414.6,448.7) | 109 (105,114) | 490.8 (471.4,509.8) | 15.7 (15.3,16.1) |
| Papua New Guinea | 10158 (9738,10545) | 322.9 (309.6,334.9) | 31615 (30415,32822) | 368.3 (354.7,382.6) | 29.5 (26.4,32.2) |
| Samoa | 497 (479,516) | 386.1 (371.8,400.2) | 806 (777,836) | 438.2 (422.5,454.7) | 13.9 (11.4,16.1) |
| Solomon Islands | 773 (742,802) | 315 (302.9,326.4) | 2038 (1958,2115) | 362.8 (349.5,375.9) | 10.2 (9.7,10.7) |
| Tokelau | 5 (5,5) | 359.8 (345.3,373.2) | 6 (6,7) | 444.1 (427.3,460.7) | 13.9 (11.6,16.3) |
| Tonga | 287 (277,297) | 369.1 (355.6,382.4) | 401 (385,415) | 427.8 (410.9,443.4) | 15.4 (13.4,17.7) |
| Tuvalu | 30 (28,31) | 356.8 (343.4,370.4) | 50 (48,52) | 428.4 (412.2,444.3) | 10.1 (7.9,12.1) |
| Vanuatu | 370 (355,384) | 330.6 (317.9,342.8) | 989 (952,1026) | 376.5 (362.5,391.5) | 7 (6.5,7.5) |
| **South Asia** | 3143242 (3043455,3246837) | 332.6 (322,342.8) | 6603080 (6392737,6815308) | 361 (349.5,372.4) | 5.3 (3.4,7.2) |
| Bangladesh | 265585 (255658,275330) | 303 (292.5,313.7) | 576489 (557167,596920) | 352.5 (341.1,364.4) | 22.2 (19.7,24.8) |
| Bhutan | 1474 (1416,1530) | 286.5 (276.4,296.7) | 2585 (2487,2682) | 340.5 (328.5,352.8) | 7.7 (5.7,9.8) |
| India | 2564832 (2483644,2651113) | 342 (331.1,352.7) | 5216089 (5050769,5385384) | 365.8 (354.3,377.3) | 20.9 (18.1,23.2) |
| Nepal | 42571 (41088,44083) | 262.5 (253.3,271.9) | 101457 (97750,105363) | 339.9 (327.9,352.4) | 20 (17.6,22.4) |
| Pakistan | 268780 (259688,277905) | 294.1 (284.3,303.8) | 706459 (681703,732164) | 336.4 (325.4,347.8) | 19.2 (16.8,21.4) |
| **Southeast Asia** | 1741466 (1683853,1799524) | 451.9 (437,466.8) | 3949656 (3809325,4084197) | 545.1 (526.4,563.2) | 14.6 (12.8,16.4) |
| Cambodia | 29315 (28254,30402) | 384.2 (370.4,399.1) | 74440 (71638,77363) | 469.1 (451.7,487.3) | 27.3 (24.6,29.7) |
| Indonesia | 706922 (683317,729989) | 460.2 (445,475.1) | 1613493 (1559130,1667709) | 560.6 (542.2,578.2) | 12.4 (10.9,14.1) |
| Lao People's Democratic Republic | 12069 (11625,12536) | 377.3 (363.9,392.9) | 31123 (29953,32292) | 470.8 (453.1,487.7) | 25.5 (22.8,28) |
| Malaysia | 73808 (70957,76675) | 511 (491.3,531.3) | 196569 (189067,204024) | 609.9 (586.9,632.1) | 9.8 (7.4,12.3) |
| Maldives | 671 (645,697) | 420 (404.1,436.4) | 2915 (2792,3047) | 556.8 (535,579.2) | 13.8 (13.2,14.4) |
| Mauritius | 4755 (4572,4942) | 481.8 (464.2,499.9) | 8795 (8445,9115) | 568.8 (547.4,589.6) | 10.9 (10.1,11.7) |
| Myanmar | 127950 (123099,132773) | 380.2 (366,394.8) | 265165 (254408,275934) | 475.3 (456,493.2) | 20.6 (19.9,21.2) |
| Philippines | 231768 (223825,239191) | 469.1 (453.6,483.8) | 562035 (543414,580355) | 532.3 (515,549) | 15.2 (13,17.4) |
| Seychelles | 340 (327,352) | 524.1 (505,544.2) | 711 (681,739) | 605.8 (582.7,628.9) | 15 (12.6,17.3) |
| Sri Lanka | 74870 (71851,77763) | 492.5 (473.1,510.1) | 145081 (139392,150999) | 585.8 (563.8,609.5) | 14.4 (13,15.9) |
| Thailand | 239706 (230514,248579) | 469.9 (452.5,486.3) | 476545 (458133,494877) | 556.8 (536.2,577.9) | 20.8 (18.4,23.1) |
| Timor-Leste | 2261 (2170,2352) | 392.1 (376.6,407.2) | 5759 (5542,5968) | 496.8 (478,514) | 22.1 (19.5,24.6) |
| Viet Nam | 234511 (225654,243683) | 425.9 (410.7,442.5) | 561517 (537641,585401) | 529.8 (508.2,551.4) | 16.2 (13.8,18.7) |
| **Southern Latin America** | 259335 (250525,268897) | 535.3 (517,555.3) | 454728 (438288,473169) | 609.5 (587.3,633.7) | 19.8 (17.5,22.2) |
| Uruguay | 17797 (17130,18437) | 537.2 (517,556.8) | 23347 (22482,24205) | 597.9 (575.6,620.1) | 1.7 (-0.2,3.9) |
| Argentina | 175418 (169173,182185) | 537.2 (517.9,558.3) | 298118 (287043,310271) | 604.7 (581.9,629.2) | 20.1 (17.7,22.4) |
| Chile | 66108 (63616,68622) | 529.9 (509.8,548.9) | 133238 (128184,138569) | 622.8 (599.3,647.3) | 3 (0.4,6) |
| **Southern Sub-Saharan Africa** | 101562 (98049,105128) | 229.7 (222.1,237.2) | 188239 (181892,194701) | 243.9 (236.2,252.1) | 10.6 (8.1,13) |
| Botswana | 2277 (2187,2368) | 215.9 (207.8,224.1) | 5654 (5431,5891) | 247.2 (237.6,256.6) | 5.3 (4.6,6) |
| Eswatini | 1286 (1235,1340) | 207.8 (199.8,215.8) | 2358 (2263,2457) | 232.5 (224.1,241.2) | 3.4 (1.8,5.1) |
| Lesotho | 2468 (2370,2565) | 194.1 (186.4,201.5) | 3658 (3518,3806) | 216.3 (208.5,224.8) | 10.8 (8.6,12.9) |
| Namibia | 2517 (2413,2616) | 220.5 (212,228.7) | 5311 (5097,5533) | 241.4 (232.1,250.5) | 12.5 (10.4,14.7) |
| South Africa | 75792 (73154,78435) | 235.9 (228.1,243.7) | 142746 (138100,147794) | 250.6 (242.6,259.2) | 12.1 (9.9,14.3) |
| Zimbabwe | 17223 (16540,17897) | 213.5 (205.9,221.2) | 28513 (27408,29768) | 217.1 (209.2,225.3) | 7.6 (5.6,9.6) |
| **Tropical Latin America** | 568589 (550164,586982) | 415.6 (402.4,429) | 1000423 (968021,1033148) | 410.8 (397.9,424) | 51.1 (48,54.3) |
| Brazil | 554871 (536838,572869) | 415.9 (402.7,429.4) | 971497 (940062,1003123) | 410.7 (397.8,423.8) | 7.3 (5.2,9.6) |
| Paraguay | 13718 (13242,14198) | 402.2 (388,416) | 28926 (27963,30042) | 413.8 (399.9,428.9) | 5.3 (4.6,6.1) |
| **Western Europe** | 4633922 (4481456,4789029) | 1042.2 (1007.8,1076.7) | 6379167 (6182112,6597001) | 1155.9 (1119.4,1194.5) | 25.4 (23,28) |
| Andorra | 725 (697,753) | 1196.5 (1154.5,1240.6) | 1448 (1395,1502) | 1284.3 (1239.7,1330.8) | 3.7 (1.7,5.8) |
| Austria | 98567 (95214,102454) | 1093.9 (1056.1,1135) | 137940 (133129,142796) | 1213 (1170.1,1255.1) | 16.8 (14.6,19.3) |
| Belgium | 125287 (121073,129596) | 1074 (1037.7,1110.6) | 171655 (165865,177958) | 1206.3 (1166.2,1247.9) | -4.9 (-6.8,-2.9) |
| Cyprus | 8601 (8306,8916) | 1059.1 (1022.6,1095.6) | 20648 (19923,21434) | 1253.5 (1208.9,1297.5) | 19.3 (17,21.8) |
| Denmark | 66984 (64568,69367) | 1112 (1071.4,1149.7) | 88853 (85813,92104) | 1222.9 (1178.2,1264.2) | 16.4 (14,18.7) |
| Finland | 60384 (58208,62710) | 1042.5 (1006.7,1080.7) | 81398 (78524,84409) | 1159.3 (1121.5,1200.1) | 2.3 (0.1,4.5) |
| France | 693448 (667217,717479) | 1068 (1028.7,1104.3) | 990073 (956670,1026080) | 1213.8 (1170.5,1256.1) | 3.1 (2.7,3.5) |
| Germany | 1361441 (1315244,1411198) | 1421.5 (1373.1,1470.8) | 1766752 (1706472,1831074) | 1593.7 (1543.4,1652.1) | 25 (22.5,27.7) |
| Greece | 127791 (122824,132597) | 1060.4 (1020,1098.7) | 157385 (151646,162858) | 1193.1 (1153.8,1236.6) | 26.1 (23.5,28.9) |
| Iceland | 2758 (2662,2860) | 1052.4 (1016.8,1091.4) | 4804 (4634,4987) | 1165.9 (1125.7,1209.2) | 18.1 (15.9,20.2) |
| Ireland | 38754 (37348,40056) | 1051.4 (1012.3,1088.7) | 70636 (68064,73214) | 1211.8 (1169.7,1256.3) | 30.8 (30.2,31.5) |
| Israel | 53430 (51675,55474) | 1116.7 (1079.1,1160.3) | 122817 (118826,127041) | 1226.9 (1187.5,1269.4) | 4.5 (3.4,5.4) |
| Italy | 574002 (554343,595210) | 853 (824.1,882.8) | 716291 (691304,743397) | 910.2 (878.6,943.4) | 13.8 (11.6,16.3) |
| Luxembourg | 4989 (4816,5171) | 1117.9 (1079.9,1158) | 9825 (9461,10219) | 1241.6 (1199.6,1287.4) | 15.9 (13.6,18.3) |
| Malta | 4252 (4104,4404) | 1056.7 (1020.8,1094.4) | 6888 (6649,7156) | 1211.8 (1171.5,1256.9) | 15.6 (13.3,17.9) |
| Monaco | 479 (463,496) | 1196.1 (1152.6,1237) | 656 (631,681) | 1288.5 (1244.2,1336.3) | 8.7 (5.9,11.8) |
| Netherlands | 188620 (182524,195207) | 1117.4 (1082.4,1156.1) | 267514 (257151,277772) | 1231.9 (1186.7,1280.5) | 32.6 (30,35.2) |
| Norway | 37935 (36749,39181) | 784.3 (759,810.8) | 54113 (52359,56016) | 825.8 (799.7,854.5) | -7.6 (-9.6,-5.6) |
| Portugal | 113670 (109844,117874) | 993.2 (959.5,1029.1) | 158343 (152596,164258) | 1142.4 (1101,1184.5) | 4.2 (2.1,6.2) |
| San Marino | 321 (310,334) | 1169.1 (1128.5,1213.7) | 536 (516,556) | 1257.8 (1213.3,1301) | 7.9 (5.8,9.9) |
| Spain | 292923 (282611,303253) | 672.3 (648.5,697.6) | 426379 (415499,439628) | 734 (715.2,756.4) | -5.3 (-7.2,-3.3) |
| Sweden | 81030 (78061,83817) | 803 (775.3,830.4) | 104275 (100752,108242) | 830.2 (802.7,860.5) | 7.3 (5,9.7) |
| Switzerland | 96791 (93317,100238) | 1204.3 (1163.6,1247.1) | 146598 (140949,152012) | 1299.1 (1250.3,1345.1) | 16.9 (14.6,19) |
| United Kingdom | 596932 (578999,615094) | 908.8 (880.6,936.7) | 867723 (839922,895505) | 1046.7 (1013,1080.3) | 18.5 (16.4,20.9) |
| **Western Sub-Saharan Africa** | 480572 (464508,497021) | 316.6 (306.3,327.1) | 1434639 (1385778,1484214) | 366.3 (354.8,378.5) | 30.9 (30.3,31.6) |
| Benin | 6962 (6695,7241) | 188.3 (181.8,194.9) | 21653 (20879,22458) | 201.5 (194.9,208.8) | 9.2 (6,12.6) |
| Burkina Faso | 13004 (12507,13521) | 175.1 (169.2,181.3) | 34066 (32836,35346) | 188 (181.4,195.1) | 20.7 (18,23.4) |
| Cabo Verde | 565 (545,585) | 195.7 (188.9,202.7) | 1257 (1212,1304) | 224.5 (216.8,232.3) | 15.2 (14.7,15.7) |
| Cameroon | 16893 (16301,17495) | 205.8 (198.6,212.7) | 58628 (56456,60884) | 224.8 (217,232.8) | 7.4 (5.4,9.5) |
| Chad | 8025 (7724,8303) | 171.6 (165.3,177.4) | 23871 (22926,24802) | 179.5 (173.6,185.6) | 9.2 (7,11.7) |
| Côte d'Ivoire | 18517 (17765,19253) | 196.1 (189.1,203.2) | 49021 (47152,51013) | 211.4 (204,218.9) | 4.6 (2.6,6.8) |
| Gambia | 1439 (1383,1494) | 189.6 (182.9,196.2) | 4058 (3903,4214) | 206.3 (199.4,213.5) | 7 (4.9,9.5) |
| Ghana | 22991 (21636,24265) | 191.5 (181.6,201.9) | 63166 (59673,66651) | 212 (201.1,222.9) | 14.7 (12.3,17.1) |
| Guinea | 8925 (8606,9248) | 183.1 (176.5,189.7) | 21272 (20460,22103) | 197 (189.8,203.5) | 8.8 (6.5,11.1) |
| Guinea-Bissau | 1462 (1412,1514) | 186.9 (180.6,193.1) | 3341 (3215,3474) | 200.6 (193.8,207.4) | 6.8 (4.8,9) |
| Liberia | 3736 (3608,3879) | 190.9 (184.3,197.6) | 9382 (9016,9705) | 203.8 (196.3,210.7) | 5.7 (3.6,8) |
| Mali | 12723 (12006,13413) | 186.1 (176.1,196.2) | 37544 (35471,39656) | 202.3 (191.6,213.2) | 11.1 (9,13.5) |
| Mauritania | 3280 (3164,3404) | 198.4 (191.5,205.4) | 7988 (7690,8291) | 217.6 (209.6,225.2) | 7.3 (5.3,9.4) |
| Niger | 10016 (9635,10399) | 165.2 (159.5,170.8) | 31127 (29971,32256) | 166.2 (160.7,171.4) | 9.7 (7.4,11.9) |
| Nigeria | 328727 (317592,339953) | 453.1 (438.6,467.9) | 1010164 (975218,1044865) | 551.1 (533.7,569.5) | 9.3 (6.9,11.6) |
| Sao Tome and Principe | 202 (195,209) | 207.5 (200.3,214.4) | 444 (429,460) | 230.5 (222.9,238.4) | 0.6 (-1.5,2.8) |
| Senegal | 11227 (10816,11653) | 189 (182,196) | 27754 (26746,28878) | 206.7 (199.8,213.9) | 7.6 (5.4,10) |
| Sierra Leone | 6365 (6143,6602) | 189.4 (183,195.5) | 14760 (14226,15327) | 200.2 (193.6,206.9) | 10.7 (7.7,14.1) |
| Togo | 5497 (5282,5708) | 197.3 (190.5,204.1) | 15125 (14584,15688) | 210.6 (203.2,217.6) | 21.6 (21.2,22.1) |

**Table S2. The EAPC of prevalence, incidence and DALYs for psoriasis in 1990-2019 and 2019-2021 among 204 countries.**

| **Country** | **EPAC Prevalence** | | **EPAC Incidence** | | **EPAC DALYs** | |
| --- | --- | --- | --- | --- | --- | --- |
|  | **1990-2019** | **2019-2021** | **1990-2019** | **2019-2021** | **1990-2019** | **2019-2021** |
| **Bolivia (Plurinational State of)** | 0.6 (0.58,0.63) | 0.55 (0.51,0.6) | 0.44 (0.42,0.46) | 0.3 (0.25,0.36) | 0.62 (0.6,0.64) | 0.29 (-0.16,0.74) |
| **Ecuador** | 0.56 (0.54,0.57) | 0.48 (0.44,0.52) | 0.42 (0.41,0.44) | 0.26 (0.2,0.33) | 0.56 (0.54,0.57) | 0.33 (0.22,0.45) |
| **Peru** | 0.64 (0.6,0.69) | 0.46 (0.41,0.51) | 0.48 (0.45,0.51) | 0.24 (0.23,0.24) | 0.66 (0.61,0.7) | 0.39 (0.23,0.56) |
| **Australia** | 0.66 (0.59,0.73) | 0.3 (0.25,0.34) | 0.46 (0.38,0.53) | 1.33 (0.98,1.67) | 0.65 (0.58,0.73) | 0.21 (0.13,0.29) |
| **New Zealand** | 0.35 (0.33,0.36) | 0.37 (0.31,0.43) | 0.27 (0.22,0.32) | 1.39 (0.93,1.86) | 0.37 (0.35,0.38) | 0.37 (0.34,0.4) |
| **Antigua and Barbuda** | 0.37 (0.35,0.39) | 0.24 (0.18,0.29) | 0.28 (0.27,0.3) | 0.13 (-0.03,0.29) | 0.36 (0.34,0.38) | -0.02 (-0.17,0.13) |
| **Bahamas** | 0.25 (0.23,0.27) | 0.06 (0.01,0.12) | 0.21 (0.19,0.23) | 0.03 (-0.06,0.12) | 0.24 (0.22,0.26) | -0.07 (-0.12,-0.02) |
| **Barbados** | 0.11 (0.1,0.12) | 0.05 (0.04,0.05) | 0.12 (0.11,0.13) | 0.01 (-0.05,0.07) | 0.1 (0.09,0.11) | 0.13 (0.07,0.19) |
| **Belize** | 0.4 (0.38,0.42) | 0.13 (0.09,0.17) | 0.31 (0.28,0.33) | 0.02 (-0.03,0.06) | 0.39 (0.37,0.41) | 0.22 (0.11,0.33) |
| **Bermuda** | 0.29 (0.28,0.31) | 0.14 (0.1,0.17) | 0.24 (0.23,0.25) | 0.04 (-0.11,0.19) | 0.3 (0.28,0.32) | -0.01 (-0.15,0.13) |
| **Cuba** | 0.12 (0.09,0.15) | 0.26 (0.23,0.3) | 0.13 (0.11,0.15) | 0.1 (0.01,0.18) | 0.12 (0.1,0.15) | 0.18 (0.18,0.18) |
| **Dominica** | 0.4 (0.38,0.42) | 0.17 (0.12,0.23) | 0.3 (0.28,0.32) | 0.07 (-0.06,0.2) | 0.4 (0.38,0.41) | -0.08 (-0.08,-0.08) |
| **Dominican Republic** | 0.42 (0.4,0.44) | 0.4 (0.33,0.47) | 0.32 (0.31,0.33) | 0.21 (0.17,0.25) | 0.41 (0.39,0.43) | 0.35 (0.12,0.58) |
| **Grenada** | 0.49 (0.45,0.53) | 0.27 (0.22,0.32) | 0.36 (0.33,0.39) | 0.12 (-0.02,0.26) | 0.48 (0.44,0.52) | 0.19 (-0.02,0.4) |
| **Guyana** | 0.38 (0.37,0.39) | 0.74 (0.63,0.86) | 0.29 (0.27,0.31) | 0.44 (0.41,0.48) | 0.38 (0.36,0.4) | 0.63 (0.42,0.84) |
| **Haiti** | 0.27 (0.26,0.27) | 0.17 (0.12,0.22) | 0.22 (0.21,0.24) | 0.06 (0.02,0.09) | 0.28 (0.27,0.29) | -0.13 (-0.21,-0.05) |
| **Jamaica** | 0.31 (0.29,0.32) | 0.18 (0.13,0.24) | 0.24 (0.23,0.26) | 0.09 (-0.01,0.18) | 0.3 (0.29,0.32) | -0.01 (-0.05,0.04) |
| **Puerto Rico** | 0.34 (0.33,0.35) | 0.26 (0.2,0.32) | 0.27 (0.25,0.29) | 0.11 (-0.02,0.25) | 0.34 (0.32,0.35) | 0.2 (-0.08,0.48) |
| **Saint Kitts and Nevis** | 0.36 (0.35,0.38) | 0.25 (0.24,0.25) | 0.28 (0.27,0.3) | 0.11 (0.08,0.13) | 0.37 (0.35,0.38) | 0.2 (0.05,0.35) |
| **Saint Lucia** | 0.4 (0.38,0.43) | 0.13 (0.07,0.18) | 0.31 (0.28,0.33) | 0.06 (-0.04,0.16) | 0.41 (0.38,0.43) | -0.11 (-0.51,0.3) |
| **Saint Vincent and the Grenadines** | 0.36 (0.34,0.38) | 0.25 (0.24,0.26) | 0.28 (0.26,0.3) | 0.14 (0.04,0.24) | 0.35 (0.34,0.37) | 0.07 (-0.31,0.46) |
| **Suriname** | 0.3 (0.29,0.32) | 0.17 (0.14,0.19) | 0.24 (0.23,0.25) | 0.08 (-0.01,0.17) | 0.29 (0.28,0.31) | -0.14 (-0.21,-0.08) |
| **Trinidad and Tobago** | 0.37 (0.35,0.39) | 0.1 (0.04,0.16) | 0.28 (0.27,0.3) | 0.05 (-0.08,0.18) | 0.36 (0.34,0.38) | -0.08 (-0.24,0.08) |
| **United States Virgin Islands** | 0.28 (0.26,0.29) | 0.14 (0.07,0.21) | 0.23 (0.21,0.24) | 0.05 (-0.12,0.21) | 0.26 (0.24,0.28) | 0.15 (-0.08,0.38) |
| **Armenia** | 0.6 (0.56,0.64) | 0.61 (0.57,0.64) | 0.48 (0.45,0.52) | 0.47 (0.17,0.78) | 0.61 (0.57,0.65) | 0.32 (0.04,0.61) |
| **Azerbaijan** | 0.44 (0.37,0.5) | 0.65 (0.6,0.7) | 0.37 (0.33,0.41) | 0.51 (0.2,0.81) | 0.45 (0.38,0.51) | 0.49 (0.37,0.61) |
| **Georgia** | 0.22 (0.17,0.27) | 0.6 (0.58,0.62) | 0.22 (0.19,0.26) | 0.43 (0.2,0.66) | 0.2 (0.16,0.25) | 0.41 (0.04,0.78) |
| **Kazakhstan** | 0.44 (0.42,0.45) | 0.59 (0.51,0.67) | 0.37 (0.35,0.39) | 0.42 (0.09,0.76) | 0.44 (0.41,0.46) | 0.51 (0.35,0.67) |
| **Kyrgyzstan** | 0.21 (0.18,0.24) | 0.63 (0.55,0.72) | 0.22 (0.2,0.24) | 0.45 (0.11,0.8) | 0.22 (0.18,0.25) | 0.48 (0.18,0.79) |
| **Mongolia** | 0.49 (0.47,0.52) | 0.64 (0.56,0.71) | 0.41 (0.38,0.44) | 0.47 (0.2,0.74) | 0.49 (0.46,0.52) | 0.46 (0.25,0.67) |
| **Tajikistan** | 0.28 (0.21,0.35) | 0.7 (0.55,0.85) | 0.28 (0.23,0.32) | 0.5 (0.2,0.8) | 0.28 (0.21,0.35) | 0.55 (-0.27,1.38) |
| **Turkmenistan** | 0.42 (0.38,0.47) | 0.76 (0.71,0.81) | 0.37 (0.34,0.4) | 0.49 (0.16,0.82) | 0.43 (0.39,0.47) | 0.78 (0.58,0.99) |
| **Uzbekistan** | 0.51 (0.49,0.53) | 0.72 (0.62,0.81) | 0.43 (0.41,0.45) | 0.53 (0.21,0.85) | 0.52 (0.5,0.54) | 0.64 (0.37,0.92) |
| **Albania** | 0.59 (0.56,0.63) | 0.54 (0.41,0.67) | 0.48 (0.45,0.51) | 0.33 (0.09,0.58) | 0.62 (0.58,0.65) | 0.19 (0.17,0.22) |
| **Bosnia and Herzegovina** | 0.84 (0.77,0.91) | 0.5 (0.34,0.67) | 0.62 (0.57,0.68) | 0.26 (0.01,0.51) | 0.84 (0.77,0.91) | 0.25 (0.18,0.33) |
| **Bulgaria** | 0.49 (0.47,0.51) | 0.49 (0.35,0.62) | 0.4 (0.38,0.42) | 0.29 (0.08,0.51) | 0.5 (0.48,0.52) | 0.16 (-0.17,0.48) |
| **Croatia** | 0.52 (0.5,0.55) | 0.44 (0.34,0.54) | 0.43 (0.4,0.45) | 0.21 (-0.14,0.56) | 0.53 (0.5,0.55) | 0.28 (0.22,0.33) |
| **Czechia** | 0.48 (0.43,0.53) | 0.25 (0.15,0.35) | 0.4 (0.36,0.43) | 0.11 (-0.1,0.31) | 0.48 (0.43,0.52) | 0.42 (0.08,0.76) |
| **Hungary** | 0.52 (0.48,0.56) | 0.55 (0.35,0.76) | 0.42 (0.38,0.45) | 0.32 (0.1,0.55) | 0.55 (0.51,0.59) | 0.5 (0.13,0.88) |
| **Montenegro** | 0.49 (0.45,0.53) | 0.45 (0.26,0.64) | 0.4 (0.37,0.43) | 0.26 (0.05,0.46) | 0.48 (0.44,0.52) | 0.3 (0.16,0.43) |
| **North Macedonia** | 0.55 (0.53,0.58) | 0.49 (0.39,0.59) | 0.44 (0.42,0.47) | 0.25 (-0.03,0.53) | 0.56 (0.53,0.58) | 0.42 (0.06,0.78) |
| **Poland** | 0.35 (0.33,0.38) | 0.3 (0.28,0.31) | 0.31 (0.28,0.33) | 0.14 (-0.23,0.51) | 0.38 (0.36,0.41) | 0.13 (0.01,0.25) |
| **Romania** | 0.43 (0.41,0.46) | 0.48 (0.15,0.81) | 0.37 (0.35,0.4) | 0.28 (0.12,0.44) | 0.46 (0.43,0.49) | 0.39 (0.06,0.72) |
| **Serbia** | 0.58 (0.55,0.61) | 0.52 (0.38,0.66) | 0.45 (0.43,0.48) | 0.29 (0.06,0.51) | 0.59 (0.55,0.62) | 0.57 (0.17,0.96) |
| **Slovakia** | 0.56 (0.52,0.59) | 0.22 (0.11,0.34) | 0.44 (0.41,0.48) | 0.1 (-0.19,0.4) | 0.58 (0.55,0.62) | 0.08 (-0.22,0.38) |
| **Slovenia** | 0.47 (0.43,0.5) | 0.43 (0.28,0.57) | 0.39 (0.36,0.42) | 0.24 (0.03,0.44) | 0.48 (0.45,0.51) | 0.17 (0.14,0.21) |
| **Colombia** | 0.35 (0.32,0.38) | 0.37 (0.36,0.37) | 0.28 (0.25,0.3) | 0.16 (0.09,0.24) | 0.37 (0.34,0.4) | 0.26 (-0.04,0.56) |
| **Costa Rica** | 0.32 (0.31,0.32) | 0.21 (0.18,0.25) | 0.25 (0.24,0.27) | 0.04 (-0.01,0.09) | 0.31 (0.3,0.32) | 0.01 (-0.19,0.21) |
| **El Salvador** | 0.48 (0.45,0.51) | 0.27 (0.21,0.34) | 0.36 (0.34,0.38) | 0.13 (0.07,0.19) | 0.49 (0.46,0.51) | 0.11 (0,0.22) |
| **Guatemala** | 0.54 (0.52,0.56) | 0.39 (0.35,0.44) | 0.4 (0.38,0.41) | 0.22 (0.17,0.27) | 0.54 (0.52,0.56) | 0.24 (0.03,0.46) |
| **Honduras** | 0.43 (0.42,0.44) | 0.34 (0.32,0.36) | 0.33 (0.32,0.34) | 0.18 (0.17,0.2) | 0.42 (0.41,0.43) | 0.16 (0.1,0.22) |
| **Mexico** | 0.31 (0.31,0.31) | 0.24 (0.21,0.27) | 0.25 (0.24,0.27) | 0.09 (0.05,0.14) | 0.31 (0.31,0.32) | 0.1 (0.06,0.14) |
| **Nicaragua** | 0.41 (0.39,0.44) | 0.18 (0.1,0.26) | 0.32 (0.29,0.34) | 0.11 (0.02,0.2) | 0.42 (0.39,0.44) | 0.01 (-0.21,0.22) |
| **Panama** | 0.29 (0.27,0.3) | 0.39 (0.35,0.44) | 0.23 (0.22,0.25) | 0.15 (0.1,0.2) | 0.28 (0.26,0.29) | 0.39 (0.19,0.59) |
| **Venezuela (Bolivarian Republic of)** | 0.18 (0.14,0.21) | -0.1 (-0.15,-0.05) | 0.17 (0.13,0.2) | -0.13 (-0.25,-0.01) | 0.18 (0.14,0.21) | -0.22 (-0.47,0.04) |
| **Angola** | 0.78 (0.72,0.83) | 0.67 (0.6,0.73) | 0.62 (0.57,0.67) | 0.29 (0.18,0.41) | 0.8 (0.74,0.86) | 0.47 (0.42,0.52) |
| **Central African Republic** | 0.32 (0.3,0.34) | 0.32 (0.15,0.48) | 0.31 (0.29,0.33) | 0.08 (0.02,0.15) | 0.35 (0.33,0.37) | 0.32 (0.11,0.52) |
| **Congo** | 0.59 (0.55,0.63) | 0.45 (0.39,0.5) | 0.48 (0.44,0.52) | 0.17 (0.07,0.27) | 0.6 (0.56,0.65) | 0.38 (0.2,0.57) |
| **Democratic Republic of the Congo** | 0.29 (0.18,0.4) | 0.54 (0.47,0.61) | 0.28 (0.2,0.37) | 0.29 (0.17,0.42) | 0.34 (0.22,0.46) | 0.46 (0.45,0.47) |
| **Equatorial Guinea** | 1.61 (1.54,1.67) | 0.48 (0.44,0.52) | 1.17 (1.12,1.21) | 0.21 (0.02,0.4) | 1.66 (1.58,1.74) | 0.33 (0.29,0.37) |
| **Gabon** | 0.75 (0.74,0.76) | 0.54 (0.48,0.61) | 0.59 (0.58,0.6) | 0.24 (0.11,0.37) | 0.76 (0.75,0.77) | 0.24 (0.09,0.38) |
| **China** | 0.91 (0.86,0.96) | 0.59 (0.57,0.62) | 0.77 (0.73,0.8) | 0.57 (0.47,0.66) | 0.92 (0.87,0.96) | 0.55 (0.55,0.56) |
| **Democratic People's Republic of Korea** | 0.63 (0.61,0.65) | 0.54 (0.54,0.54) | 0.59 (0.58,0.61) | 0.52 (0.44,0.59) | 0.64 (0.61,0.66) | 0.44 (0.4,0.48) |
| **Taiwan (Province of China)** | 0.94 (0.83,1.06) | 1.84 (1.83,1.84) | 0.84 (0.76,0.93) | 1.49 (1.41,1.58) | 0.93 (0.82,1.05) | 1.76 (1.59,1.93) |
| **Belarus** | 0.53 (0.5,0.56) | 0.64 (0.56,0.72) | 0.43 (0.4,0.46) | 0.46 (0.14,0.78) | 0.54 (0.51,0.57) | 0.36 (-0.04,0.76) |
| **Estonia** | 0.58 (0.55,0.62) | 0.56 (0.4,0.73) | 0.46 (0.43,0.5) | 0.38 (-0.03,0.79) | 0.59 (0.55,0.63) | 0.5 (0.11,0.89) |
| **Latvia** | 0.51 (0.48,0.55) | 0.57 (0.42,0.72) | 0.42 (0.38,0.45) | 0.42 (0.06,0.79) | 0.54 (0.5,0.58) | 0.36 (0.35,0.37) |
| **Lithuania** | 0.66 (0.63,0.68) | 0.72 (0.63,0.82) | 0.51 (0.48,0.54) | 0.52 (0.09,0.96) | 0.67 (0.64,0.7) | 0.39 (0.37,0.4) |
| **Republic of Moldova** | 0.48 (0.44,0.52) | 0.7 (0.53,0.87) | 0.4 (0.37,0.42) | 0.51 (0.09,0.94) | 0.5 (0.46,0.54) | 0.43 (-0.26,1.13) |
| **Russian Federation** | 0.44 (0.42,0.46) | 0.66 (0.52,0.79) | 0.37 (0.35,0.39) | 0.48 (0.11,0.86) | 0.46 (0.43,0.49) | 0.45 (0.28,0.61) |
| **Ukraine** | 0.35 (0.32,0.38) | 0.51 (0.46,0.56) | 0.31 (0.28,0.33) | 0.35 (0.05,0.65) | 0.36 (0.33,0.4) | 0.26 (-0.03,0.54) |
| **Burundi** | -0.14 (-0.15,-0.13) | -0.42 (-0.88,0.03) | -0.06 (-0.07,-0.05) | -0.37 (-0.8,0.07) | -0.12 (-0.13,-0.11) | -0.66 (-0.99,-0.33) |
| **Comoros** | 0.14 (0.12,0.15) | -0.23 (-0.77,0.31) | 0.15 (0.15,0.16) | -0.22 (-0.69,0.25) | 0.15 (0.14,0.17) | -0.42 (-0.92,0.09) |
| **Djibouti** | 0.08 (0.07,0.1) | 0.01 (-0.36,0.39) | 0.11 (0.09,0.13) | -0.07 (-0.39,0.24) | 0.09 (0.07,0.11) | 0.15 (-0.24,0.54) |
| **Eritrea** | 0.14 (0.11,0.17) | -0.17 (-0.6,0.26) | 0.15 (0.14,0.17) | -0.2 (-0.61,0.22) | 0.16 (0.13,0.19) | -0.25 (-0.38,-0.12) |
| **Ethiopia** | 0.16 (0.13,0.19) | 0.06 (-0.05,0.18) | 0.17 (0.14,0.2) | -0.03 (-0.07,0.02) | 0.2 (0.17,0.23) | -0.19 (-0.23,-0.15) |
| **Kenya** | 0.09 (0.07,0.1) | -0.01 (-0.44,0.41) | 0.11 (0.09,0.12) | -0.09 (-0.47,0.29) | 0.11 (0.09,0.13) | -0.22 (-0.49,0.06) |
| **Madagascar** | -0.05 (-0.07,-0.03) | -0.05 (-0.55,0.44) | 0.01 (-0.01,0.03) | -0.1 (-0.56,0.35) | -0.02 (-0.04,0) | -0.02 (-0.18,0.15) |
| **Malawi** | 0.12 (0.09,0.15) | -0.1 (-0.56,0.36) | 0.14 (0.11,0.16) | -0.15 (-0.55,0.26) | 0.16 (0.13,0.19) | -0.3 (-0.48,-0.12) |
| **Mozambique** | 0.14 (0.12,0.16) | -0.04 (-0.53,0.46) | 0.15 (0.13,0.17) | -0.06 (-0.5,0.38) | 0.15 (0.12,0.17) | -0.5 (-0.94,-0.06) |
| **Rwanda** | 0.19 (0.16,0.21) | -0.47 (-1.41,0.48) | 0.19 (0.16,0.21) | -0.45 (-1.31,0.43) | 0.19 (0.16,0.23) | -0.63 (-1.46,0.2) |
| **Somalia** | -0.24 (-0.24,-0.23) | -0.44 (-0.93,0.06) | -0.16 (-0.17,-0.16) | -0.44 (-0.88,0.01) | -0.21 (-0.22,-0.2) | -0.73 (-1.45,0) |
| **South Sudan** | -0.12 (-0.14,-0.09) | -0.39 (-0.94,0.17) | -0.04 (-0.06,-0.02) | -0.35 (-0.88,0.18) | -0.1 (-0.12,-0.08) | -0.57 (-0.76,-0.39) |
| **Uganda** | 0.31 (0.28,0.33) | 0.06 (-0.44,0.57) | 0.28 (0.25,0.3) | -0.02 (-0.41,0.37) | 0.33 (0.3,0.36) | -0.08 (-0.2,0.04) |
| **United Republic of Tanzania** | 0.11 (0.1,0.12) | -0.03 (-0.63,0.57) | 0.13 (0.12,0.15) | -0.1 (-0.57,0.38) | 0.16 (0.14,0.17) | -0.62 (-0.78,-0.47) |
| **Zambia** | 0.18 (0.14,0.22) | -0.05 (-0.55,0.45) | 0.18 (0.15,0.22) | -0.12 (-0.56,0.32) | 0.18 (0.13,0.23) | -0.05 (-0.23,0.14) |
| **Brunei Darussalam** | 0.25 (0.23,0.26) | 0.4 (0.31,0.5) | 0.2 (0.15,0.25) | 1.63 (0.93,2.34) | 0.24 (0.22,0.26) | 0.36 (0.18,0.54) |
| **Japan** | 0.03 (0.01,0.05) | 0.34 (0.19,0.48) | 0.04 (0,0.09) | 1.49 (0.98,1.99) | 0.04 (0.01,0.06) | 0.27 (0.16,0.38) |
| **Republic of Korea** | 0.4 (0.36,0.44) | 0.43 (0.29,0.57) | 0.3 (0.25,0.36) | 1.57 (0.89,2.26) | 0.42 (0.38,0.46) | 0.39 (0.33,0.44) |
| **Singapore** | 0.42 (0.38,0.45) | 0.2 (0.02,0.38) | 0.31 (0.25,0.37) | 1.53 (0.91,2.15) | 0.43 (0.39,0.47) | 0.23 (0.06,0.39) |
| **Canada** | 0.39 (0.38,0.39) | 0.2 (0.13,0.27) | 0.27 (0.22,0.32) | 1.43 (0.61,2.26) | 0.38 (0.37,0.38) | 0.25 (-0.06,0.55) |
| **Greenland** | 0.49 (0.46,0.51) | 0.35 (0.32,0.38) | 0.33 (0.28,0.38) | 1.55 (0.57,2.53) | 0.5 (0.47,0.52) | 0.15 (0,0.3) |
| **United States of America** | 0.13 (0.1,0.15) | -0.17 (-0.46,0.11) | 0.08 (0.04,0.13) | 1.14 (0.65,1.63) | 0.1 (0.08,0.12) | -0.38 (-0.62,-0.15) |
| **Afghanistan** | 0.48 (0.43,0.54) | 0.59 (0.5,0.68) | 0.47 (0.44,0.5) | 0.41 (0.37,0.45) | 0.47 (0.41,0.53) | 0.5 (0.49,0.5) |
| **Algeria** | 0.62 (0.61,0.64) | 0.39 (0.35,0.43) | 0.56 (0.54,0.57) | 0.26 (0.21,0.31) | 0.63 (0.61,0.65) | 0.31 (-0.05,0.67) |
| **Bahrain** | 0.66 (0.64,0.69) | 0.25 (0.2,0.29) | 0.58 (0.56,0.61) | 0.22 (0.22,0.23) | 0.66 (0.64,0.69) | 0.12 (-0.15,0.39) |
| **Egypt** | 0.72 (0.68,0.75) | 0.61 (0.45,0.77) | 0.64 (0.62,0.67) | 0.43 (0.34,0.53) | 0.73 (0.69,0.77) | 0.48 (0.28,0.69) |
| **Iran (Islamic Republic of)** | 0.84 (0.8,0.88) | 0.32 (0.29,0.34) | 0.69 (0.66,0.72) | 0.21 (0.14,0.28) | 0.84 (0.8,0.88) | 0.15 (0.02,0.28) |
| **Iraq** | 0.91 (0.88,0.93) | 0.72 (0.63,0.81) | 0.74 (0.73,0.75) | 0.51 (0.45,0.57) | 0.91 (0.88,0.93) | 0.43 (0.11,0.75) |
| **Jordan** | 0.62 (0.6,0.64) | 0.36 (0.34,0.38) | 0.55 (0.54,0.57) | 0.27 (0.27,0.27) | 0.61 (0.59,0.63) | 0.24 (0.21,0.28) |
| **Kuwait** | 0.64 (0.62,0.65) | 0.34 (0.27,0.41) | 0.56 (0.55,0.57) | 0.23 (0.19,0.28) | 0.61 (0.59,0.63) | 0.24 (-0.01,0.49) |
| **Lebanon** | 0.76 (0.75,0.77) | 0.31 (0.29,0.33) | 0.65 (0.64,0.66) | 0.21 (0.19,0.23) | 0.75 (0.74,0.76) | 0.01 (-0.01,0.02) |
| **Libya** | 0.84 (0.75,0.94) | -0.68 (-0.69,-0.67) | 0.69 (0.62,0.76) | -0.36 (-0.39,-0.34) | 0.81 (0.72,0.91) | -0.82 (-1.2,-0.44) |
| **Morocco** | 0.6 (0.58,0.62) | 0.61 (0.54,0.69) | 0.54 (0.53,0.55) | 0.45 (0.44,0.45) | 0.59 (0.57,0.61) | 0.37 (0.2,0.55) |
| **Oman** | 1.04 (0.99,1.09) | 0.35 (0.34,0.36) | 0.82 (0.79,0.86) | 0.3 (0.28,0.32) | 1.05 (1,1.1) | 0.26 (0.22,0.29) |
| **Palestine** | 0.77 (0.75,0.79) | 0.6 (0.58,0.61) | 0.65 (0.65,0.66) | 0.38 (0.37,0.38) | 0.76 (0.73,0.78) | 0.32 (0.29,0.35) |
| **Qatar** | 0.68 (0.64,0.72) | 0.61 (0.55,0.66) | 0.6 (0.57,0.63) | 0.42 (0.42,0.43) | 0.67 (0.63,0.71) | 0.62 (0.55,0.69) |
| **Saudi Arabia** | 0.98 (0.96,1.01) | 0.43 (0.42,0.45) | 0.78 (0.76,0.8) | 0.32 (0.26,0.38) | 0.97 (0.95,1) | 0.32 (0.22,0.43) |
| **Sudan** | 0.92 (0.89,0.94) | 0.64 (0.64,0.65) | 0.75 (0.74,0.76) | 0.4 (0.34,0.46) | 0.93 (0.9,0.95) | 0.49 (0.49,0.49) |
| **Syrian Arab Republic** | 0.86 (0.82,0.89) | 0.39 (0.38,0.39) | 0.7 (0.67,0.73) | 0.23 (0.22,0.24) | 0.84 (0.81,0.88) | 0.04 (0.01,0.06) |
| **Tunisia** | 0.74 (0.71,0.77) | 0.36 (0.35,0.36) | 0.63 (0.6,0.65) | 0.26 (0.23,0.29) | 0.73 (0.7,0.76) | 0 (-0.08,0.08) |
| **Turkey** | 0.93 (0.91,0.96) | 0.51 (0.45,0.57) | 0.76 (0.74,0.77) | 0.36 (0.34,0.37) | 0.94 (0.92,0.96) | 0.37 (0.26,0.48) |
| **United Arab Emirates** | 0.74 (0.68,0.79) | 0.3 (0.17,0.43) | 0.63 (0.59,0.68) | 0.19 (0.09,0.29) | 0.73 (0.68,0.79) | 0.2 (-0.05,0.45) |
| **Yemen** | 0.87 (0.84,0.9) | 0.26 (0.25,0.27) | 0.72 (0.69,0.74) | 0.2 (0.2,0.2) | 0.87 (0.84,0.9) | 0.34 (0.28,0.4) |
| **American Samoa** | 0.34 (0.33,0.35) | 0.23 (0.01,0.46) | 0.31 (0.29,0.32) | 0.28 (0.14,0.42) | 0.31 (0.3,0.32) | 0.22 (-0.19,0.63) |
| **Cook Islands** | 0.48 (0.47,0.49) | 0.37 (0.16,0.59) | 0.41 (0.4,0.41) | 0.4 (0.23,0.56) | 0.47 (0.45,0.48) | 0.08 (-0.2,0.35) |
| **Fiji** | 0.49 (0.48,0.5) | 0.3 (0.12,0.48) | 0.43 (0.42,0.44) | 0.29 (0.16,0.41) | 0.48 (0.47,0.49) | 0.07 (-0.05,0.18) |
| **Guam** | 0.5 (0.45,0.55) | 0.14 (-0.07,0.35) | 0.42 (0.39,0.45) | 0.18 (0.06,0.31) | 0.49 (0.45,0.54) | 0.12 (0.05,0.2) |
| **Kiribati** | 0.39 (0.39,0.4) | 0.37 (0.15,0.58) | 0.36 (0.35,0.37) | 0.32 (0.22,0.41) | 0.41 (0.39,0.42) | 0.21 (-0.07,0.48) |
| **Marshall Islands** | 0.4 (0.38,0.41) | 0.43 (0.28,0.57) | 0.35 (0.33,0.36) | 0.45 (0.34,0.56) | 0.38 (0.36,0.4) | 0.38 (0.01,0.75) |
| **Micronesia (Federated States of)** | 0.45 (0.44,0.45) | 0.3 (0.18,0.42) | 0.4 (0.39,0.4) | 0.32 (0.27,0.37) | 0.44 (0.43,0.45) | 0.37 (0.31,0.43) |
| **Nauru** | 0.34 (0.26,0.42) | 0.61 (0.39,0.84) | 0.33 (0.27,0.39) | 0.54 (0.49,0.59) | 0.32 (0.24,0.41) | 0.48 (-0.13,1.08) |
| **Niue** | 0.58 (0.56,0.59) | 0.28 (0.06,0.49) | 0.49 (0.48,0.5) | 0.26 (0.09,0.43) | 0.57 (0.56,0.58) | 0.41 (0.19,0.63) |
| **Northern Mariana Islands** | 0.16 (0.12,0.2) | 0.18 (0.03,0.33) | 0.18 (0.16,0.21) | 0.29 (0.28,0.3) | 0.16 (0.11,0.2) | 0.08 (-0.03,0.19) |
| **Palau** | 0.41 (0.38,0.43) | 0.13 (-0.08,0.34) | 0.38 (0.36,0.4) | 0.22 (0.14,0.3) | 0.39 (0.36,0.41) | 0.18 (0.05,0.31) |
| **Papua New Guinea** | 0.4 (0.39,0.41) | 0.27 (0.14,0.4) | 0.36 (0.35,0.37) | 0.29 (0.15,0.44) | 0.42 (0.4,0.43) | 0.34 (0.12,0.57) |
| **Samoa** | 0.42 (0.41,0.42) | 0.27 (0.09,0.45) | 0.38 (0.38,0.39) | 0.25 (0.19,0.31) | 0.41 (0.4,0.42) | 0.26 (0.06,0.45) |
| **Solomon Islands** | 0.43 (0.4,0.45) | 0.31 (0.11,0.52) | 0.39 (0.38,0.41) | 0.3 (0.28,0.32) | 0.42 (0.4,0.45) | 0.25 (0.15,0.34) |
| **Tokelau** | 0.7 (0.69,0.72) | 0.42 (0.2,0.63) | 0.59 (0.58,0.61) | 0.36 (0.26,0.46) | 0.68 (0.66,0.7) | 0.25 (0.17,0.32) |
| **Tonga** | 0.45 (0.44,0.46) | 0.39 (0.14,0.63) | 0.41 (0.4,0.41) | 0.36 (0.28,0.44) | 0.44 (0.43,0.45) | 0.09 (-0.3,0.47) |
| **Tuvalu** | 0.56 (0.55,0.57) | 0.52 (0.23,0.8) | 0.51 (0.5,0.52) | 0.49 (0.32,0.66) | 0.58 (0.57,0.59) | 0.32 (0.2,0.45) |
| **Vanuatu** | 0.43 (0.41,0.44) | 0.24 (0.07,0.42) | 0.38 (0.37,0.39) | 0.27 (0.12,0.42) | 0.43 (0.42,0.44) | 0.12 (0.08,0.16) |
| **Bangladesh** | 0.47 (0.46,0.48) | 0.53 (0.49,0.57) | 0.41 (0.4,0.42) | 0.48 (0.36,0.6) | 0.49 (0.47,0.5) | 0.38 (0.13,0.62) |
| **Bhutan** | 0.61 (0.6,0.62) | 0.36 (0.31,0.4) | 0.5 (0.49,0.5) | 0.36 (0.17,0.54) | 0.62 (0.61,0.64) | 0.53 (0.33,0.73) |
| **India** | -0.04 (-0.1,0.02) | 3.54 (-0.23,7.45) | 0.08 (0.05,0.11) | 2.34 (0.12,4.6) | -0.02 (-0.08,0.04) | 3.39 (-0.35,7.26) |
| **Nepal** | 0.67 (0.53,0.81) | 0.78 (0.7,0.85) | 0.55 (0.46,0.64) | 0.61 (0.46,0.76) | 0.7 (0.56,0.84) | 0.65 (0.27,1.04) |
| **Pakistan** | 0.43 (0.42,0.45) | 0.49 (0.45,0.54) | 0.39 (0.38,0.4) | 0.42 (0.33,0.51) | 0.44 (0.42,0.45) | 0.34 (0.13,0.56) |
| **Cambodia** | 0.69 (0.67,0.71) | 0.39 (-0.02,0.8) | 0.57 (0.56,0.59) | 0.38 (0.09,0.68) | 0.72 (0.7,0.74) | 0.21 (-0.34,0.77) |
| **Indonesia** | 0.62 (0.6,0.64) | 0.32 (-0.07,0.71) | 0.5 (0.49,0.51) | 0.3 (0.09,0.51) | 0.63 (0.61,0.65) | 0.21 (-0.28,0.7) |
| **Lao People's Democratic Republic** | 0.75 (0.73,0.77) | 0.49 (0.08,0.91) | 0.6 (0.59,0.62) | 0.41 (0.22,0.59) | 0.79 (0.76,0.81) | 0.48 (0.25,0.72) |
| **Malaysia** | 0.61 (0.57,0.65) | 0.09 (-0.24,0.42) | 0.5 (0.47,0.52) | 0.16 (-0.04,0.36) | 0.62 (0.58,0.65) | 0.01 (-0.12,0.14) |
| **Maldives** | 0.95 (0.9,0.99) | 0.36 (0.11,0.61) | 0.77 (0.75,0.79) | 0.45 (0.35,0.55) | 0.97 (0.93,1.01) | 0.59 (0.49,0.68) |
| **Mauritius** | 0.54 (0.53,0.55) | 0.16 (-0.15,0.48) | 0.45 (0.45,0.46) | 0.18 (0.03,0.33) | 0.53 (0.51,0.54) | -0.01 (-0.29,0.28) |
| **Myanmar** | 0.78 (0.76,0.8) | 0.45 (0.08,0.82) | 0.61 (0.6,0.63) | 0.4 (0.2,0.61) | 0.81 (0.78,0.83) | 0.36 (-0.14,0.86) |
| **Philippines** | 0.37 (0.34,0.39) | 0.38 (-0.01,0.77) | 0.34 (0.32,0.36) | 0.34 (0.13,0.55) | 0.39 (0.36,0.41) | 0.26 (-0.1,0.63) |
| **Seychelles** | 0.44 (0.41,0.47) | 0.15 (-0.18,0.48) | 0.38 (0.36,0.4) | 0.21 (0.06,0.36) | 0.43 (0.39,0.46) | 0.06 (-0.61,0.73) |
| **Sri Lanka** | 0.56 (0.55,0.58) | 0.23 (-0.15,0.61) | 0.47 (0.46,0.48) | 0.24 (0.03,0.46) | 0.56 (0.54,0.58) | 0.08 (-0.12,0.29) |
| **Thailand** | 0.54 (0.53,0.56) | 0.18 (-0.19,0.54) | 0.45 (0.44,0.46) | 0.2 (-0.06,0.45) | 0.56 (0.54,0.58) | 0.15 (0.03,0.26) |
| **Timor-Leste** | 0.89 (0.86,0.92) | 0.09 (-0.28,0.46) | 0.7 (0.68,0.72) | 0.14 (-0.06,0.34) | 0.92 (0.88,0.95) | 0.09 (-0.05,0.23) |
| **Viet Nam** | 0.74 (0.73,0.74) | 0.41 (-0.03,0.85) | 0.6 (0.59,0.6) | 0.38 (0.17,0.58) | 0.76 (0.75,0.77) | 0.37 (-0.08,0.82) |
| **Uruguay** | 0.34 (0.33,0.35) | 0.36 (0.32,0.4) | 0.25 (0.2,0.3) | 1.47 (0.92,2.03) | 0.33 (0.32,0.34) | 0.11 (-0.04,0.26) |
| **Argentina** | 0.33 (0.32,0.35) | 0.43 (0.41,0.45) | 0.25 (0.2,0.3) | 1.53 (0.9,2.18) | 0.33 (0.31,0.34) | 0.52 (0.52,0.52) |
| **Chile** | 0.51 (0.5,0.52) | 0.44 (0.43,0.46) | 0.36 (0.31,0.41) | 1.53 (0.97,2.09) | 0.51 (0.5,0.52) | 0.41 (0.37,0.45) |
| **Botswana** | 0.52 (0.5,0.54) | -0.29 (-0.68,0.1) | 0.45 (0.43,0.47) | -0.32 (-0.53,-0.12) | 0.5 (0.47,0.52) | -0.54 (-0.73,-0.34) |
| **Eswatini** | 0.43 (0.42,0.45) | -0.22 (-0.67,0.24) | 0.39 (0.37,0.41) | -0.3 (-0.61,0.01) | 0.37 (0.35,0.38) | -0.26 (-0.8,0.28) |
| **Lesotho** | 0.43 (0.42,0.45) | -0.28 (-0.66,0.1) | 0.39 (0.37,0.41) | -0.3 (-0.53,-0.07) | 0.36 (0.34,0.38) | -0.49 (-0.58,-0.41) |
| **Namibia** | 0.37 (0.34,0.4) | -0.31 (-0.75,0.13) | 0.35 (0.32,0.38) | -0.34 (-0.67,-0.01) | 0.36 (0.32,0.4) | -0.46 (-0.94,0.03) |
| **South Africa** | 0.3 (0.27,0.33) | -0.42 (-0.82,-0.02) | 0.3 (0.27,0.32) | -0.4 (-0.64,-0.16) | 0.27 (0.24,0.3) | -0.65 (-0.93,-0.37) |
| **Zimbabwe** | 0.03 (0,0.06) | -0.46 (-0.94,0.03) | 0.1 (0.08,0.12) | -0.45 (-0.75,-0.14) | 0.02 (-0.01,0.05) | -0.62 (-1.3,0.05) |
| **Brazil** | -0.07 (-0.08,-0.05) | -0.02 (-0.03,0) | -0.01 (-0.03,0.01) | -0.07 (-0.14,0.01) | -0.05 (-0.07,-0.03) | -0.21 (-0.24,-0.18) |
| **Paraguay** | 0.07 (0.06,0.08) | 0.1 (0.08,0.11) | 0.09 (0.07,0.1) | 0.02 (-0.01,0.05) | 0.06 (0.05,0.07) | -0.07 (-0.11,-0.02) |
| **Andorra** | 0.24 (0.23,0.24) | 0.24 (0.15,0.33) | 0.19 (0.15,0.24) | 1.09 (0.24,1.94) | 0.23 (0.23,0.24) | 0.12 (-0.06,0.3) |
| **Austria** | 0.34 (0.33,0.35) | 0.22 (0.14,0.31) | 0.26 (0.21,0.3) | 1.07 (0.31,1.83) | 0.35 (0.34,0.36) | 0.26 (-0.04,0.56) |
| **Belgium** | 0.37 (0.36,0.38) | 0.28 (0.23,0.33) | 0.28 (0.23,0.32) | 1.11 (0.45,1.79) | 0.36 (0.35,0.37) | 0.3 (0.12,0.49) |
| **Cyprus** | 0.56 (0.52,0.6) | 0.25 (0.13,0.38) | 0.41 (0.34,0.47) | 1.04 (0.25,1.84) | 0.57 (0.53,0.61) | 0.18 (0.16,0.21) |
| **Denmark** | 0.31 (0.3,0.32) | 0.25 (0.14,0.36) | 0.24 (0.19,0.28) | 1.09 (0.33,1.86) | 0.33 (0.31,0.34) | 0.27 (-0.17,0.71) |
| **Finland** | 0.35 (0.34,0.36) | 0.29 (0.17,0.42) | 0.27 (0.22,0.31) | 1.1 (0.29,1.91) | 0.35 (0.35,0.36) | 0.35 (0.34,0.36) |
| **France** | 0.38 (0.32,0.44) | 0.34 (0.17,0.5) | 0.29 (0.22,0.36) | 1.13 (0.36,1.91) | 0.38 (0.33,0.44) | 0.24 (0.12,0.36) |
| **Germany** | 0.33 (0.25,0.42) | 0.54 (0.31,0.78) | 0.26 (0.18,0.34) | 1.22 (0.65,1.8) | 0.33 (0.25,0.42) | 0.48 (0.35,0.62) |
| **Greece** | 0.39 (0.36,0.42) | 0.24 (0.14,0.35) | 0.29 (0.24,0.35) | 1.07 (0.34,1.81) | 0.39 (0.37,0.41) | 0.2 (-0.02,0.42) |
| **Iceland** | 0.32 (0.31,0.33) | 0.27 (0.18,0.35) | 0.25 (0.21,0.3) | 1.09 (0.32,1.87) | 0.33 (0.33,0.34) | 0.11 (-0.19,0.41) |
| **Ireland** | 0.46 (0.44,0.48) | 0.33 (0.27,0.38) | 0.34 (0.29,0.39) | 1.13 (0.52,1.75) | 0.47 (0.45,0.49) | 0.27 (0.13,0.42) |
| **Israel** | 0.3 (0.28,0.31) | 0.23 (0.19,0.27) | 0.24 (0.19,0.28) | 1.08 (0.38,1.79) | 0.3 (0.29,0.32) | 0.16 (0.03,0.28) |
| **Italy** | 0.15 (0.12,0.19) | 0.28 (0.23,0.33) | 0.13 (0.08,0.18) | 1.12 (0.42,1.82) | 0.17 (0.14,0.21) | 0.16 (0,0.32) |
| **Luxembourg** | 0.33 (0.32,0.34) | 0.34 (0.26,0.42) | 0.25 (0.2,0.3) | 1.13 (0.41,1.85) | 0.34 (0.34,0.35) | 0.21 (0.09,0.33) |
| **Malta** | 0.44 (0.43,0.45) | 0.41 (0.33,0.49) | 0.32 (0.28,0.37) | 1.19 (0.41,1.97) | 0.44 (0.43,0.45) | 0.32 (0.1,0.54) |
| **Monaco** | 0.24 (0.23,0.24) | 0.26 (0.12,0.4) | 0.19 (0.15,0.24) | 1.06 (0.28,1.84) | 0.23 (0.23,0.24) | 0.17 (-0.11,0.45) |
| **Netherlands** | 0.31 (0.3,0.32) | 0.24 (0.18,0.3) | 0.24 (0.19,0.29) | 1.08 (0.35,1.81) | 0.31 (0.3,0.33) | -0.03 (-0.3,0.23) |
| **Norway** | 0.18 (0.15,0.22) | 0.14 (0.13,0.15) | 0.16 (0.1,0.22) | 0.98 (0.36,1.61) | 0.21 (0.18,0.24) | 0.03 (-0.07,0.14) |
| **Portugal** | 0.45 (0.43,0.46) | 0.33 (0.31,0.35) | 0.33 (0.28,0.38) | 1.11 (0.47,1.75) | 0.46 (0.45,0.48) | 0.18 (0.14,0.23) |
| **San Marino** | 0.25 (0.23,0.27) | 0.1 (-0.05,0.24) | 0.2 (0.15,0.25) | 0.94 (0.18,1.71) | 0.23 (0.22,0.25) | -0.06 (-0.27,0.15) |
| **Spain** | 0.18 (0.12,0.24) | 0.22 (0.1,0.34) | 0.16 (0.08,0.23) | 0.97 (0.35,1.61) | 0.19 (0.13,0.24) | 0.15 (-0.02,0.32) |
| **Sweden** | 0.08 (0.05,0.1) | 0.14 (-0.06,0.34) | 0.09 (0.03,0.16) | 0.98 (0.23,1.74) | 0.08 (0.05,0.11) | 0.08 (-0.26,0.42) |
| **Switzerland** | 0.25 (0.25,0.25) | 0.17 (0.09,0.25) | 0.2 (0.16,0.25) | 1.05 (0.34,1.76) | 0.26 (0.26,0.27) | 0.07 (0.07,0.08) |
| **United Kingdom** | 0.42 (0.36,0.48) | 0.05 (-0.28,0.39) | 0.32 (0.25,0.38) | 0.86 (-0.03,1.76) | 0.42 (0.36,0.48) | -0.05 (-0.45,0.34) |
| **Benin** | 0.37 (0.32,0.41) | -0.57 (-1.11,-0.02) | 0.36 (0.32,0.41) | -0.56 (-0.87,-0.26) | 0.39 (0.35,0.44) | -0.62 (-1.02,-0.22) |
| **Burkina Faso** | 0.41 (0.36,0.47) | -0.7 (-1.32,-0.07) | 0.4 (0.35,0.45) | -0.65 (-1.07,-0.22) | 0.46 (0.4,0.52) | -0.69 (-0.97,-0.41) |
| **Cabo Verde** | 0.67 (0.61,0.72) | -0.66 (-1.24,-0.07) | 0.56 (0.51,0.61) | -0.62 (-0.97,-0.28) | 0.67 (0.61,0.72) | -0.8 (-1.55,-0.05) |
| **Cameroon** | 0.43 (0.37,0.49) | -0.54 (-1.06,-0.01) | 0.4 (0.35,0.46) | -0.51 (-0.85,-0.17) | 0.45 (0.39,0.52) | -0.89 (-1.42,-0.37) |
| **Chad** | 0.33 (0.27,0.39) | -0.73 (-1.18,-0.27) | 0.34 (0.28,0.39) | -0.64 (-0.97,-0.3) | 0.34 (0.28,0.4) | -1.06 (-1.27,-0.84) |
| **C?te d'Ivoire** | 0.37 (0.33,0.42) | -0.47 (-0.85,-0.1) | 0.37 (0.32,0.41) | -0.44 (-0.58,-0.3) | 0.42 (0.37,0.46) | -0.53 (-0.92,-0.14) |
| **Gambia** | 0.45 (0.39,0.5) | -0.6 (-1.12,-0.07) | 0.42 (0.37,0.47) | -0.55 (-0.89,-0.21) | 0.44 (0.39,0.5) | -0.77 (-1.26,-0.28) |
| **Ghana** | 0.54 (0.48,0.61) | -0.61 (-1.06,-0.17) | 0.48 (0.42,0.54) | -0.59 (-0.85,-0.33) | 0.57 (0.5,0.64) | -0.83 (-1.29,-0.37) |
| **Guinea** | 0.38 (0.33,0.43) | -0.52 (-1.11,0.08) | 0.37 (0.33,0.42) | -0.53 (-0.85,-0.21) | 0.4 (0.36,0.45) | -0.67 (-1.56,0.23) |
| **Guinea-Bissau** | 0.38 (0.34,0.43) | -0.64 (-1.26,-0.02) | 0.37 (0.33,0.42) | -0.59 (-0.97,-0.21) | 0.4 (0.36,0.44) | -0.74 (-1.12,-0.36) |
| **Liberia** | 0.45 (0.38,0.53) | -0.71 (-1.11,-0.31) | 0.42 (0.35,0.49) | -0.62 (-0.87,-0.38) | 0.48 (0.39,0.56) | -0.72 (-0.74,-0.69) |
| **Mali** | 0.53 (0.46,0.6) | -0.99 (-1.64,-0.33) | 0.47 (0.41,0.53) | -0.82 (-1.23,-0.41) | 0.55 (0.48,0.62) | -0.9 (-1.57,-0.24) |
| **Mauritania** | 0.46 (0.4,0.51) | -0.6 (-1.18,-0.02) | 0.42 (0.37,0.47) | -0.56 (-0.82,-0.3) | 0.48 (0.43,0.53) | -0.76 (-1.59,0.08) |
| **Niger** | 0.17 (0.11,0.22) | -0.76 (-1.33,-0.18) | 0.22 (0.17,0.27) | -0.7 (-1.09,-0.31) | 0.2 (0.14,0.26) | -0.63 (-1.32,0.05) |
| **Nigeria** | 0.75 (0.69,0.8) | 0.35 (0.17,0.53) | 0.6 (0.54,0.65) | 0.05 (-0.01,0.12) | 0.78 (0.71,0.84) | 0.23 (-0.1,0.56) |
| **Sao Tome and Principe** | 0.56 (0.49,0.63) | -0.63 (-1.16,-0.09) | 0.49 (0.42,0.55) | -0.57 (-0.79,-0.34) | 0.57 (0.5,0.65) | -0.72 (-1.55,0.11) |
| **Senegal** | 0.43 (0.38,0.48) | -0.51 (-1.03,0.02) | 0.41 (0.36,0.45) | -0.5 (-0.85,-0.15) | 0.45 (0.4,0.5) | -0.57 (-1.13,0) |
| **Sierra Leone** | 0.35 (0.28,0.43) | -0.63 (-1.1,-0.16) | 0.35 (0.29,0.42) | -0.57 (-0.81,-0.32) | 0.38 (0.3,0.47) | -0.56 (-1.53,0.43) |
| **Togo** | 0.34 (0.3,0.39) | -0.62 (-1.25,0.02) | 0.35 (0.3,0.4) | -0.57 (-0.98,-0.15) | 0.35 (0.31,0.4) | -0.63 (-1.88,0.64) |
